# Selective brain entry of lipid nanoparticles in haemorrhagic stroke is linked to biphasic blood-brain barrier disruption

**DOI:** 10.7150/thno.72167

**Published:** 2022-05-26

**Authors:** Zahraa S. Al-Ahmady, Ben R. Dickie, Isabelle Aldred, Dhifaf A. Jasim, Jack Barrington, Michael Haley, Eloise Lemarchand, Graham Coutts, Satinderdeep Kaur, Jessica Bates, Sarah Curran, Ruth Goddard, Megan Walker, Adrian Parry-jones, Kostas Kostarelos, Stuart M. Allan

**Affiliations:** 1 Pharmacology Department, School of Science and Technology, Nottingham Trent University, Nottingham, NG11 8NS, United Kingdom.; 2 Nanomedicine Lab, Division of Pharmacy and Optometry, Faculty of Biology, Medicine and Health, AV Hill Building, The University of Manchester, Manchester M13 9PT, United Kingdom.; 3 Division of Neuroscience and Experimental Psychology, School of Biological Sciences, Faculty of Biology, Medicine and Health, University of Manchester, Manchester, M13 9PT, United Kingdom.; 4 Division of Cardiovascular Sciences, Lydia Becker Institute of Immunology and Inflammation, School of Medical Sciences, Faculty of Biology, Medicine and Health, Manchester Academic Health Science Centre, The University of Manchester, Manchester, United Kingdom.; 5 Manchester Centre for Clinical Neurosciences, Salford Royal NHS Foundation Trust, Manchester Academic Health Science Centre, Salford, United Kingdom.; 6 Nanomedicine Lab, Catalan Institute of Nanoscience and Nanotechnology (ICN2), Bellaterra UAB Campus, Barcelona, Spain.; 7 Geoffrey Jefferson Brain Research Centre, The Manchester Academic Health Science Centre, Northern Care Alliance NHS Group, University of Manchester, United Kingdom.

**Keywords:** Liposomes, blood-brain barrier, drug delivery, haemorrhagic stroke, lipid nanoparticles

## Abstract

Haemorrhagic stroke represents a significant public health burden, yet our knowledge and ability to treat this type of stroke are lacking. Previously we showed that we can target ischaemic-stroke lesions by selective translocation of lipid nanoparticles through the site of blood-brain barrier (BBB) disruption. The data we presented in this study provide compelling evidence that haemorrhagic stroke in mice induces BBB injury that mimics key features of the human pathology and, more importantly, provides a gate for entry of lipid nanoparticles-based therapeutics selectively to the bleeding site.

**Methods:** Haemorrhagic stroke was induced in mice by intra-striatal collagenase injection. lipid nanoparticles were injected intravenously at 3 h, 24 h & 48 h post-haemorrhagic stroke and accumulation in the brain studied using *in-vivo* optical imaging and histology. BBB integrity, brain water content and iron accumulation were characterised using dynamic contrast-enhanced MRI, quantitative T_1_ mapping, and gradient echo MRI.

**Results:** Using *in-vivo* SPECT/CT imaging and optical imaging revealed biphasic lipid nanoparticles entry into the bleeding site, with an early phase of increased uptake at 3-24 h post-haemorrhagic stroke, followed by a second phase at 48-72 h. Lipid nanoparticles entry into the brain post-haemorrhage showed an identical entry pattern to the trans-BBB leakage rate (K^trans^ [min^-1^]) of Gd-DOTA, a biomarker for BBB disruption, measured using dynamic contrast-enhanced MRI.

**Discussion:** Our findings suggest that selective accumulation of liposomes into the lesion site is linked to a biphasic pattern of BBB hyper-permeability. This approach provides a unique opportunity to selectively and efficiently deliver therapeutic molecules across the BBB, an approach that has not been utilised for haemorrhagic stroke therapy and is not achievable using free small drug molecules.

## Introduction

Stroke poses a major clinical threat, attributing to 7 % of deaths worldwide 1. It is the second cause of death globally affecting 17 million people every year, with only one-third of patients surviving with no complications 2. Stroke can be categorised into major subtypes of different pathophysiology, ischaemic and haemorrhagic stroke. Ischaemic stroke is caused by cerebrovascular vessel occlusion resulting from thrombus or emboli. Haemorrhagic stroke is caused by a rupture of a damaged or abnormal blood vessel leading to local bleeding within the cranium and accounts for 15-20 % of all stroke cases 3. Although haemorrhagic stroke has a lower incidence rate, outcomes are worse than ischaemic stroke with almost 40 % mortality risk 4. Haemorrhagic stroke can be further sub-categorised into intracerebral haemorrhage (ICH), subarachnoid and interventricular bleeds depending on the location of the ruptured vessel. The most common of those is ICH, which results from a direct leak of blood into the brain parenchyma due to vascular rupture secondary to hypertension, cerebral amyloid angiopathy, vascular aneurysm, tumour, or predisposing genetic conditions 5. Age is another un-adjustable risk factor for ICH which doubles the chance of the disease every 10 years of life 6. The increased use of oral anticoagulant to prevent ischaemic stroke causes another threat and carries a 50 % mortality rate in the occurrence of ICH due to the increased chances of re-bleeding 7,8

Secondary injury is triggered in ICH due to the breakdown of blood products and this has been shown to stimulate several cellular mechanisms which in turn exacerbate the original injury. Extracellular contact with blood components exerts neurotoxic effects from the release of free radicals causing vasogenic oedema 9. Brain-resident (microglia) and infiltrating immune cells (macrophages) attempt to clean up the haematoma debris, which is the initial driving force of the inflammatory response to stop further inflammation 10 and in parallel, a host of chemokines and cytokines are released 11. Red blood cell (RBC) lysis also occurs, triggering oxidative stress and free radical formation. Iron toxicity has also been noted to cause microglial activation and proliferation, enhancing the inflammatory response. 12. Any recovery in ICH is thought to be dependent on haematoma resolution, oedema reduction, neuroplastic abilities of the brain and neurogenesis 13.

Treatments that can salvage damaged brain tissue after ICH are currently lacking 14. The lack of treatments in ICH is partly due to the complexity of the underlying pathophysiology but also the difficulty in getting enough drug into the injury site due to the protective nature of the blood-brain barrier (BBB) 15. This highlights a real need to develop new strategies to enhance drug delivery to the brain in ICH to increase survival rates and improve the post-stroke quality of life for ICH patients. Under normal conditions, the BBB creates a sterile environment for neurons via tight control of the movement of substances between peripheral blood and neuronal tissues. This interface comprises endothelial cells and the tight junction proteins that regulate paracellular transport, pericytes, astrocytes, and transcellular vesicles which regulate the trafficking of small molecules through the endothelial cells 16,17. Maintaining BBB function is vital to maintain normal brain function with damage contributing to a host of neurological pathologies 16. Paradoxically, the protective nature of BBB also limits the delivery of therapeutic drugs into the brain. However, preclinical data 18 and also human studies 19 have shown that BBB integrity is altered following ICH, which may provide a window of opportunity to deliver drugs effectively into the haematoma and surrounding brain tissue. Therefore, understanding the mechanisms and the timeframe of BBB alteration in ICH is crucial for the development of potential new treatments.

Previously, we showed that we can selectively target the lesioned area in ischaemic stroke using lipid nanoparticles (liposomes) by taking advantage of enhanced transcellular and paracellular pathways that contribute to BBB disruption after cerebral ischaemia 20. Liposomes offer several advantages compared to conventional medications including; a) small size and long circulation half-life that enable selective and efficient lesion targeting, and b) adaptation to encapsulate a wide range of therapeutic molecules and contrast agents which offer an ideal opportunity to image where the drug is being delivered 21-23.

In this study, we hypothesised that similar selective targeting is possible using liposomes in ICH, with BBB disruption being the primary mechanism by which entrance is permitted. Such an approach provides a unique opportunity to selectively and efficiently deliver therapeutic molecules across the BBB, an approach that has not been utilised for ICH therapy and is not achievable using free small drug molecules due to short circulation half-life 24. To understand the timing of liposome penetration into the brain in ICH, we measured brain liposome accumulation using radioisotope and optical detection methods. To confirm that leakage of liposomes was primarily through a disrupted BBB, we used dynamic-contrast enhanced MRI to detect the leakage of MRI contrast agent Gd-DOTA. Similar patterns of leakage were observed strongly indicating that liposome penetrance at acute timepoints (0-72 h post-ICH) is mediated by self-diffusion across the disrupted BBB.

## Results

To establish the validity of our new therapeutic approach, we have induced ICH in mice by intracerebral injection of bacterial collagenase. This model degrades the extracellular matrix causing endothelial basement membrane destruction and subsequent haemorrhage in the brain parenchyma 25. This model offers a non-invasive and reproducible platform to mimic the pathological alterations seen clinically in ICH, including inflammatory responses and haematoma expansion, which occurs in 73 % of clinical cases 26. The other advantage of the collagenase model compared to the autologous blood model is that the collagenase model mimics the acute cerebrovascular rupture and BBB disruptions seen in ICH patients 11,27. Therefore, this model is more relevant to the aim of our study which is focused on BBB disruption, unlike the autologous blood model which is often used to study blood clotting effects.

In this study, we injected a liposome platform (HSPC:CHOL: DSPE-PEG_2000_) intravenously at different time points following ICH (3 h, 24 h & 48 h) and performed a comprehensive analysis of their accumulation in the brain and correlated this to changes in BBB integrity and ICH pathology. These liposomes are around 120 nm in size and slightly negatively charged (for full characterisation data of liposomes, refer to Figure S1).

### Liposomes accumulate at the lesion site in a biphasic pattern and maintain long term localisation after selective entry into the brain

**Figure 1:**
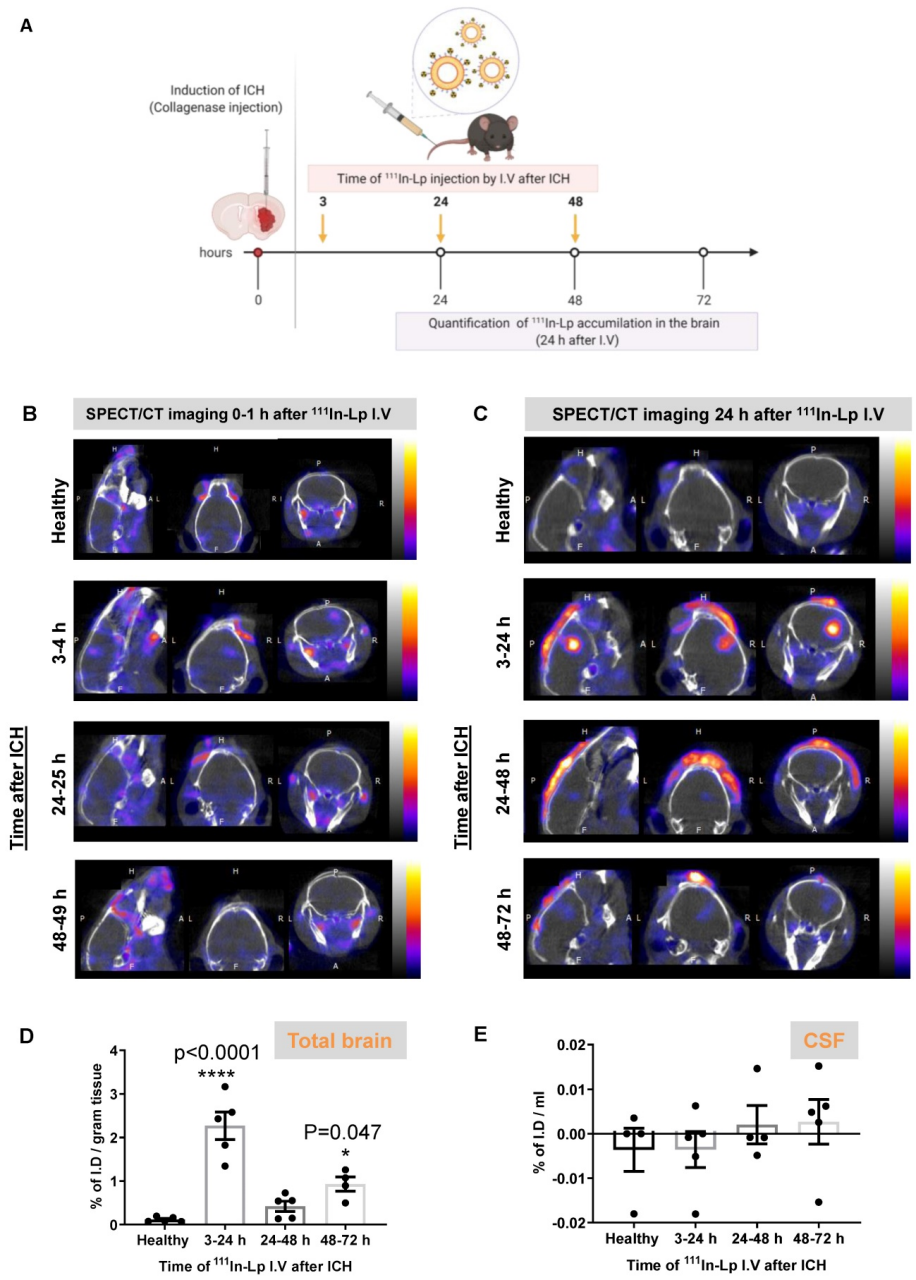
Imaging and quantification of liposomes accumulation into the brain after inducing ICH into the right hemisphere (A) Schematic presentation of experimental design and the time frame of ^111^In-Lp I.V injection after ICH. Each time point represents a separate group that received a single injection of ^111^In-Lp (8-9 MBq) I.V. Representative SPECT/CT imaging for the same mice over B) the first hour after I.V administration and C) 24 h later confirmed the selective accumulation of the liposomes into the ipsilateral side of the brain (right) compared to the contralateral side. Images shown here are for the same mouse from each group. The scale bar is the same in B and C. Healthy mice (where no ICH was induced) showed no detectable ^111^In-Lp level in the brain. The signal observed in healthy mice is due to the presence of ^111^In-Lp in the blood that is mainly found in the circulation outside the brain. This reduced substantially 24 h after injection of ^111^In-Lp as they start to clear from the blood. D) Quantification of the ^111^In-Lp level in the brain 24 h after I.V injection revealed a biphasic entry pattern with maximum accumulation observed between 3-24 h and 48-72 h post-ICH. Values are expressed as % of I.D ± SEM per gram brain tissue. (E) Quantification of ^111^In-Lp levels in the CSF indicated no significant differences in CSF liposomal level compared to healthy mice. The data in D & E were analysed by one-way ANOVA followed by Tukey multiple comparison tests (n = 4-5). The data were considered significant if p values < 0.05.

To study liposomal translocation into the brain after ICH we performed dynamic SPECT/CT imaging after I.V administration of ^111^In-DTPA-liposomes (^111^In-Lp). For full physicochemical characterisation, radiolabelling efficiency and stability see Figure S1. Following induction of ICH with collagenase, ^111^In-Lp were injected at three different time points post-ICH (3 h, 24 h and 48 h) designed to cover the different stages of ICH pathology (Figure 1A). Each injection of ^111^In-Lp was detected using SPECT-CT either 1 h (3-4 h, 24-25 h & 48-49 h, Figure 1B) or 24 h later (3-24 h, 24-48 h & 48-72 h, Figure 1C). Each time point represents a separate group that received a single injection of ^111^In-Lp I.V. Selective liposomal accumulation was seen as early as 1 h after ^111^In-Lp injection when administered acutely (3-4 h) after ICH. Repeated SPECT/CT imaging on the same animals indicated that liposomal entry into the brain increased over time with a biphasic pattern. Higher accumulation is observed both when injected at 3-24 h and 48-72 h as shown in Figure 1C. Minimum liposomal accumulation into the haemorrhagic area was observed when ^111^In-Lp were injected 24 h after ICH for both detection time points (24-25 h and 24-48 h) and no liposomal entry was observed in the contralateral side from all groups as well as healthy animals (Figure 1B-C). Quantification of ^111^In-Lp in the brain tissues 24 h after injection confirmed the significant biphasic increase in liposomes accumulation at the lesion site. An increase of 27.5-fold and 11-fold was observed at 3-24 h and 48-72 h, respectively, compared to healthy mice (Figure 1D). It is worth pointing out here, that consistent across all results we observed that the second episode of hyperpermeability was less than the first, though this is still showing to be significant. Quantification of ^111^In-Lp levels in the cerebrospinal fluid (CSF) indicated no significant differences in CSF liposomal levels compared to healthy mice. This suggested minimal clearance of liposomes after entry into the brain (Figure 1E).

The observations reported above with SPECT/CT were confirmed using fluorescently labelled liposomes coupled with optical imaging and histological analysis. For this, we injected DiI-labelled liposomes (DiI-Lp) at the same time points used for SPECT/CT imaging. Each group of mice received a single I.V injection of DiI-Lp at either 3, 24, or 48 h post-ICH. Mice were then sacrificed either 2 h after injections (3-5 h, 24-26 h & 48-50 h) or 24 h later (3-24 h, 24-48 h & 48-72 h) to evaluate the liposomal distribution in the brain and whether this changes over time.

**Figure 2:**
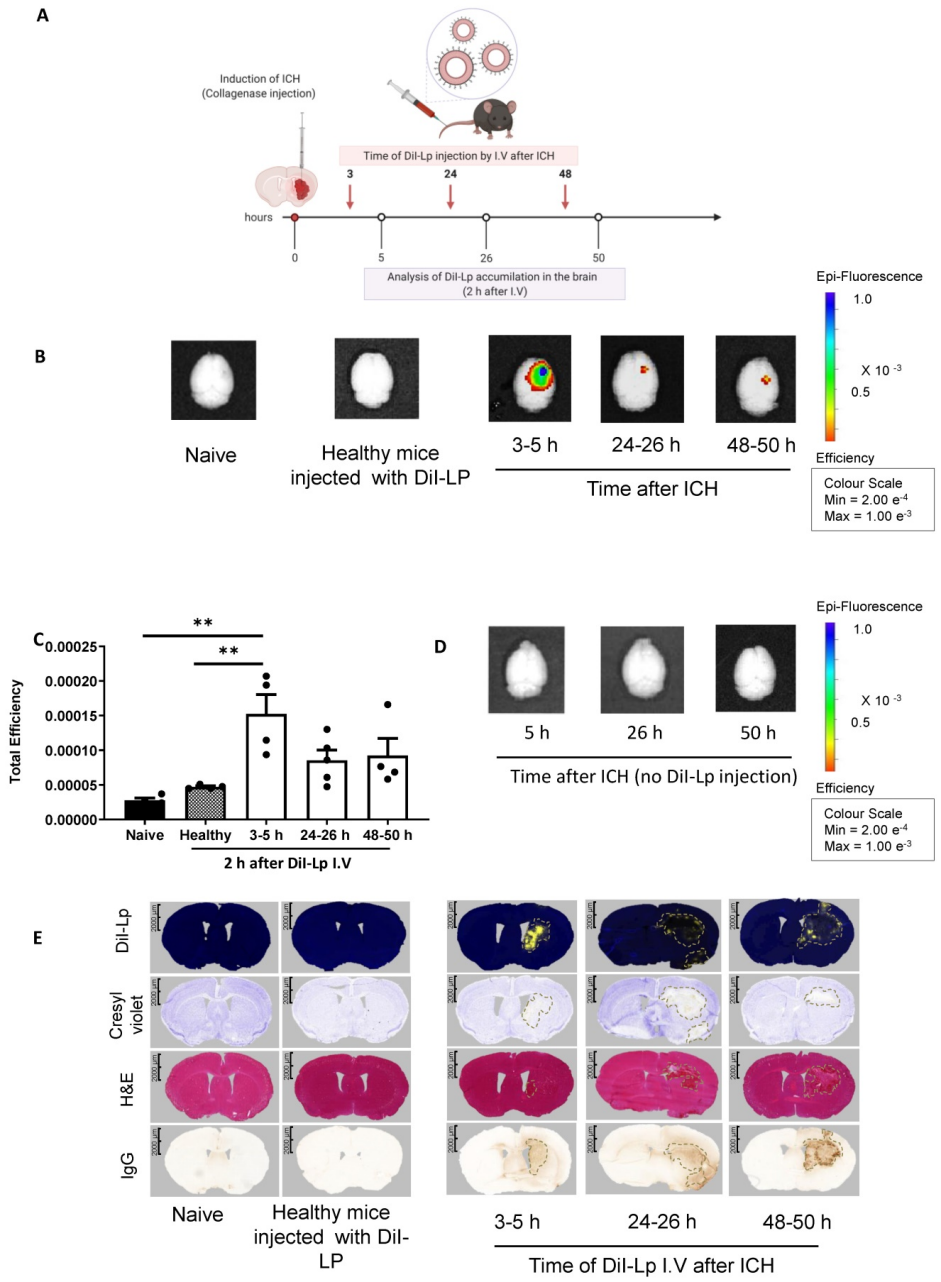
Evaluation of selective liposomal entry into the haemorrhagic brain shortly after I.V administration. A) Represents a schematic presentation of experimental design and the time frame of liposomes injection. Like the previous studies, all mice received a single I.V injection and each time point represents a separate group. B) Imaging DiI-Lp entry into the brain with IVIS Lumina II optical imaging system confirmed the selective accumulation of liposomes into the ipsilateral side (right) as early as 2 h after DiI-Lp I.V administration whereas no signal was observed on the contralateral side of the brain (left). Likewise, control groups which include naïve healthy mice injected with PBS and healthy mice injected with DiI-Lp showed no detectable signal. C) Quantification of the fluorescent signal of DiI-Lp in the brain by IVIS Lumina imaging software was performed by drawing a region of interest (ROI) that covers the whole brain and is expressed as total efficiency. Colour scale of epi-fluorescent signal range from min =2x10-4 (red) to max= 1 x10-3 (blue). Quantification data showed the significant entry of DiI-Lp into the brain only over the time frame from 3-5 h after ICH (D) to exclude any interference of tissue autofluorescence, control groups of ICH mice at a similar time point were imaged without injection of DiI-Lp which indicated no fluorescent signal in the brain. E) Histological analysis of brain tissues confirmed the presence of liposomes (yellow signal) that colocalised with the region of brain damage (represented by pale region with cresyl violet stain) and hematoma region as shown with H&E. IgG stain was performed to evaluate any endogenous IgG leakage into the brain (outline by dashed black lines) that is used as an indication of BBB disruption. Statistical analysis was performed using one-way analysis of variance followed by the Tukey multiple comparison test and p values < 0.05 were considered significant. n = 3-4 in each group.

**Figure 3:**
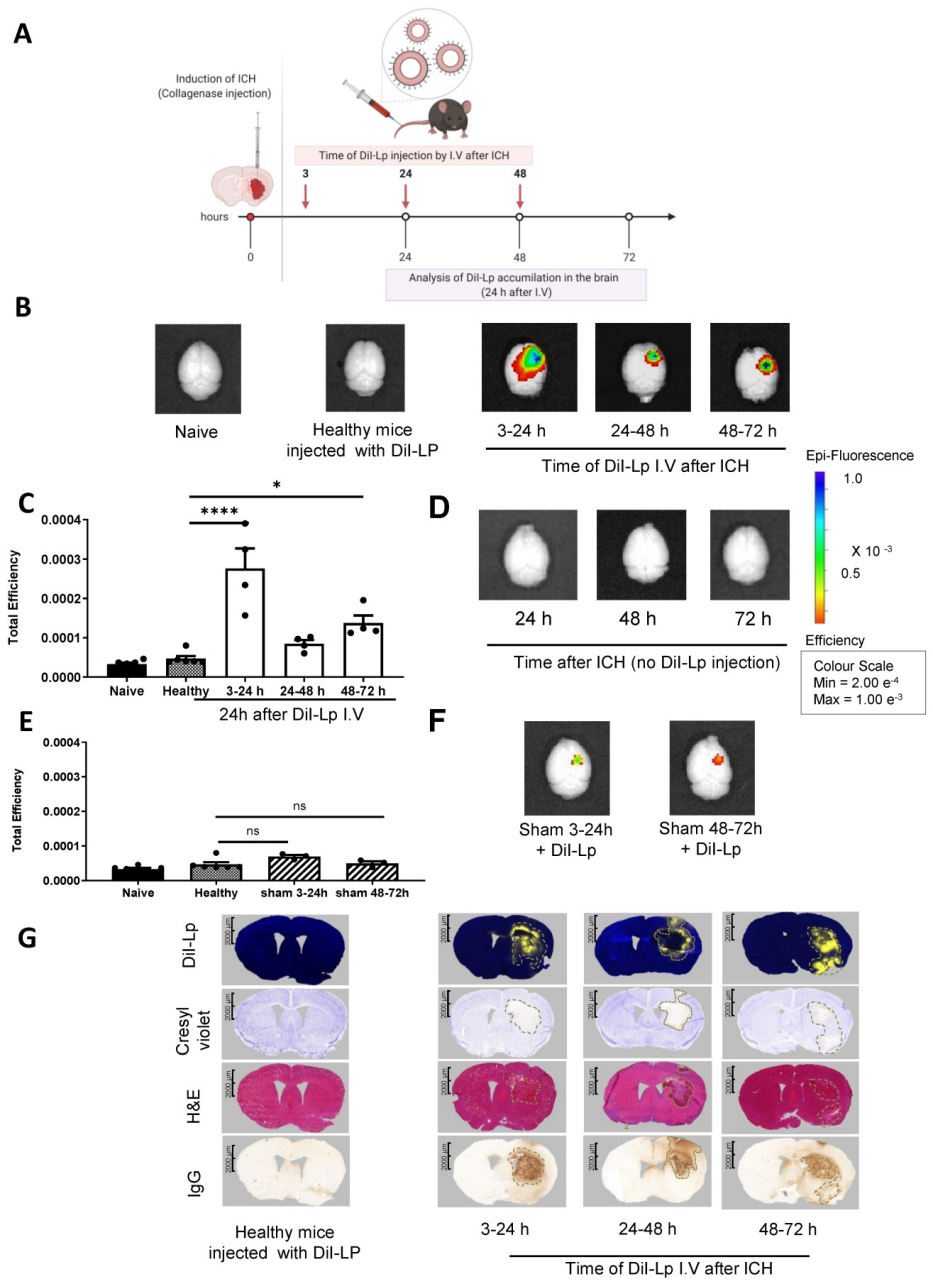
Analysis of delayed liposomal entry into the haemorrhagic brain 24 h after I.V administration. A) Schematic presentation of experimental design indicating the time frame of liposomes injection. Each time point represents a separate group of mice that received a single I.V injection. Analysis of liposomal entry into the brain was performed 24 h after I.V injection. B) IVIS Lumina II optical Imaging of DiI-Lp accumulation in the brain indicated that liposomes maintained their selective accumulation in the brain even 24 h after injection. Liposomal accumulation also revealed a biphasic entry pattern which was further confirmed by quantification of the fluorescent signal of DiI-Lp in the brain using IVIS Lumina imaging software as indicated in C). No signal was observed on the contralateral side of the brain (left) and control groups which include naïve healthy mice injected with PBS and healthy mice injected with DiI-Lp. D) IVIS images of ICH brain tissues in the absence of DiI-Lp injection did not show any fluorescent signal in the brain. E) Representative optical images of the brain after injection with DiI-Lp at the indicated time points in sham mice. F) Quantification of DiI-Lp entry from IVIS Lumina II optical images confirmed that no significant increase in liposomes entry into the brain was observed in sham mice which indicated that selective accumulation of liposomes into the ipsilateral side (right) after ICH is due to brain injury induced by ICH rather than any brain damage induced by the procedure. G) Histological analysis of brain tissues confirmed biphasic entry of liposomes (yellow signal) into the brain that colocalised with the region of brain damage (represented by pale region with cresyl violet stain) and hematoma region as shown with H&E. IgG stain was performed to evaluate any endogenous IgG leakage into the brain (outline by dashed black lines) that is used as an indication of BBB disruption. Statistical analysis was performed using one-way analysis of variance followed by the Tukey multiple comparison test and p values < 0.05 were considered significant. n = 3-4 in each group.

IVIS Lumina II imaging (Figure 2B) confirmed the selective accumulation of liposomes into the haemorrhage site (right side) of the brain as early as 2 h after DiI-Lp injection in the acute phase (3-5 h). Quantification of the total fluorescent signal of DiI-Lp in the brain 2 h after injection indicated a significant increase by 3.3-fold from the 3-5 h group compared to an increase by 1.8-fold and 2-fold detected from 24-26 h and 48-50 h groups, respectively (Figure 2B-C). In agreement with SPECT/CT images, evaluation of liposomal brain accumulation 24 h after injection revealed a biphasic entry into the brain when injected 3 h and 48 h post-ICH (Figure 3). Quantification of the optical images indicated a significant increase by 6.6-fold in the 3-24 h group and 3-fold in the 48-72 h group compared to healthy mice injected with DiI-Lp. Liposome accumulation was most prominent in the ipsilateral side, where the haemorrhage was induced. Less accumulation was observed when DiI-Lp was administered 24 h post-ICH and almost no fluorescence signal was detected in the contralateral side at all time points tested. We also found no evidence of liposomal accumulation in the brain following DiI-Lp administration in sham mice (Figure 3E-F).

### Liposomal brain entry co-localises with brain damage, haematoma, and BBB disruption

The overlap of liposomes accumulation detected from SPECT/CT and IVIS with the ipsilateral hemisphere was confirmed histologically on serial brain sections, which showed that the localisation of DiI-Lp signal is confined to the lesion site. Figure 2E shows the highest DiI-Lp accumulation in the brain when injected in the acute phase (3-5 h), staining is restricted to the infarct region with little deviation from the borderline of the haemorrhage. This indicates that liposomes are transported at the site of active bleeding through damaged vessels and preferentially reside within the area of damage. Cerebral accumulation detected from the 24-26 h group is minimal and restricted to the perihematomal region (rim area). At this time point, it can be seen that the area of infarct extends beyond the haemorrhage volume with oedema and mass effect major contributors to the observed cellular damage. At 48-50 h liposomes are seen, in a similar patterned distribution to that observed in the 24-26 h group, at the lesion rim, but also beyond the infarct region close to regions of BBB damage. The lack of liposomes within the core of the haemorrhage in the 24-26 h and 48-50 h groups likely relates to the absence of active bleeding and clot stabilisation at these timepoints.

Widespread accumulation of liposomes following injection in the acute phase (3-24 h group) is seen (Figure 3G) suggesting that they were once within the core, as shown from the 3-5 h group in Figure 2E, but that over time they disperse more widely. The distribution of liposomes injected 24 h post-ICH did not change over time and showed restricted co-localisation to the lesion rim. Interestingly, a dramatic change in liposomes distribution was observed in the 48-72 h group with DiI-Lp staining detected within the core of the haemorrhage again, as seen in the 3-24 h group, as well as the lesion rim. This is unlikely due to secondary bleeding and may be linked to the haematoma resolution process and infiltration of immune cells.

Collectively, our data identified a biphasic entry profile of liposomes into the brain after ICH with the highest accumulation observed 24 h after injection. This indicated that the long blood circulation half-life of liposomes increases the chances of extravasation into the lesion through areas of BBB disruption which is one of the advantages liposomes offer compared to small drug molecules with short circulating t_1/2_. Our previous work using a similar type of liposomes in C57 mice indicated that more than 40 % of the ID is still in circulation at 6 h after IV injection 21. Also, previous studies with other types of liposomes including drug-loaded liposomes (e.g. Doxil) indicated a circulation half-life of 1-2 days and an increase in total drug level of up to 60 folds compared to the free drug 28,29.

DiI-Lp cerebral accumulation covered both the ipsilateral cortex and striatum and maintained similar distribution to the areas of BBB damage and infarct as seen with IgG infiltration and Cresyl violet stain respectively (Figure 2E). Mice from all time points displayed altered neurological symptoms such as circling behaviours, gait and body posture that are indicative of unilateral brain damage (Figure S2A&B). All mice showed an increase in neurological score and contralateral foot-slips measures. Histological measurements of haematoma volume (Figure S2C) correlated with changes in infarct volume (Figure S2D) and body weight (Figure S2E). Changes in haematoma volume and body weight peaked at 24 h and started to reduce over time.

### Liposomes do not penetrate brain tissue in the absence of pathology

In healthy control animals, there was a lack of fluorescent signal, indicating no DiI-Lp accumulation, both with IVIS optical imaging (Figure 2B & Figure 3B) and histologically (Figure 2E and Figure 3G). Normal physiology of these animals is demonstrated with no cellular damage, absence of haemorrhage, intact BBB and hemisphere of equal sizes indicating the absence of oedema. Imaging brain tissues from animals that had ICH induction but no DiI-Lp injection also showed no increase in fluorescent signal (Figure 2D and Figure 3D), confirming that the increase in the fluorescent signal detected with IVIS is due to liposomal accumulation, and not auto-fluorescence from RBCs. Histological analysis of brain tissues of ICH mice at different time points after ICH in the absence of DiI-Lp administration confirmed the lack of autofluorescence (yellow signal) in the injury site due to the presence of RBCs in the brain (Figure S3).

### MRI revealed two windows for BBB opening that correlate with the timing of selective liposomal entry into the brain

**Figure 4:**
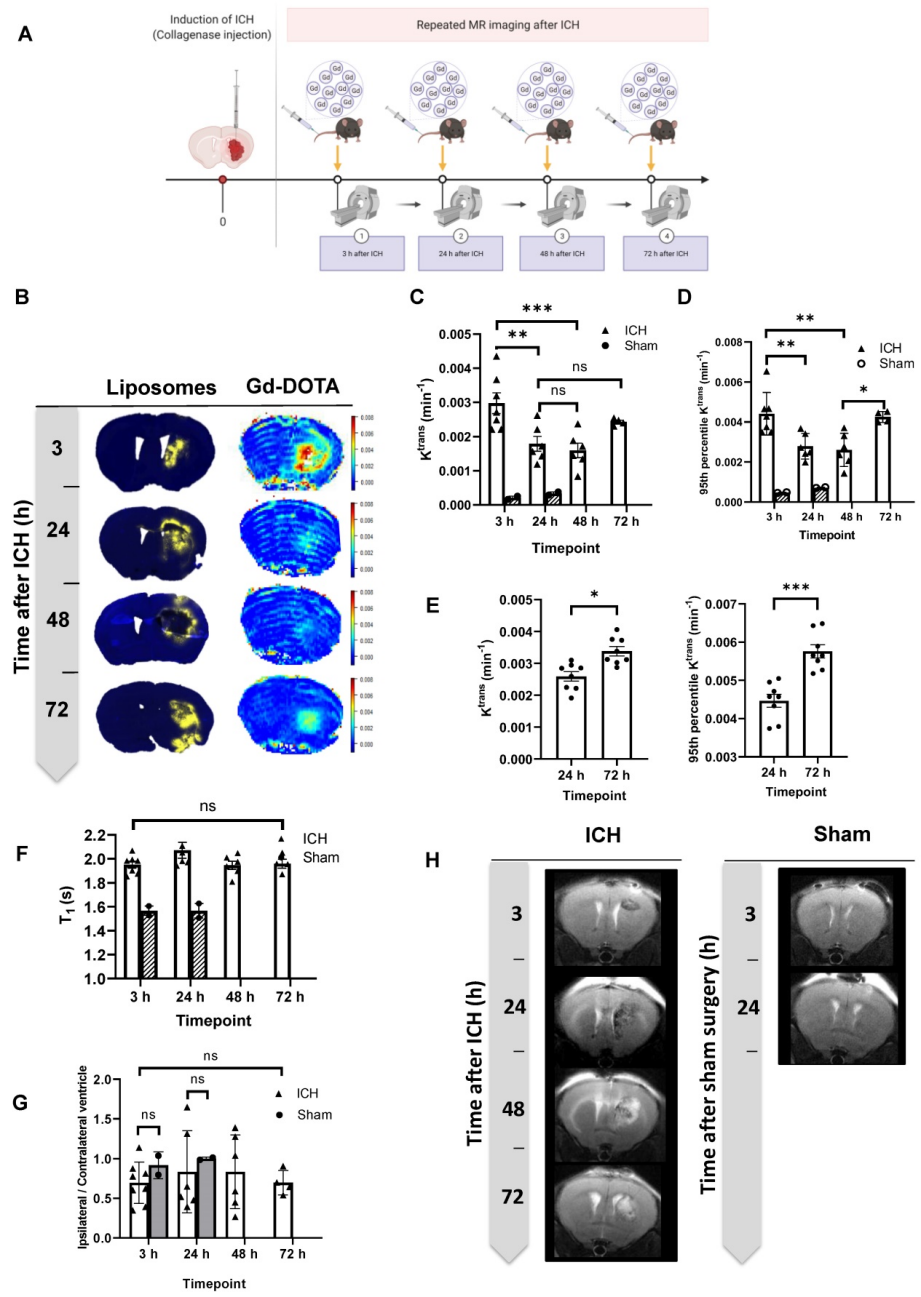
Characterisation of the blood-brain barrier and tissue changes in the haemorrhaged area by MRI at 3 h, 24 h, 48 h, and 72 h post-ICH. A) Schematic presentation of experimental design showing the time points of repeated MRI scans following ICH. 0.5 M Gd-DOTA was delivered by intravenous injection through a tail vein cannula at a volume of 3.3 µL / g at a rate of 0.25 mL / min. B) Assessment of BBB leakage was performed by measuring Gd-DOTA leakage into the brain showing similar temporal and spatial patterns to liposome accumulation, suggesting a common entry process mediated by BBB disruption. C, D & E) measurements of Gd-DOTA leakage rate (Ktrans) acquired in 2 separate cohorts (cohort 1: C, D; cohort 2: E) indicated biphasic enhancement in BBB leakage. F) Repeated measurements of spin-lattice relaxation time (T1) indicated elevated values in the ICH model (white columns) compared to sham mice (grey shaded columns). No significant changes in T1 values were observed over time post-ICH. G) analysis of T2-TurboRARE MRI for the changes in ipsilateral/contralateral ventricle ratios showed no significant differences which indicates that no brain atrophy occurs at this stage of the disease which can also be seen on H) T2 Turbo rare MR images taken at different time points after ICH and sham surgeries. Statistical analysis was performed using a one-way analysis of variance followed by the Tukey multiple comparison test and p values < 0.05 were considered significant. n = 4-7 in each group.

Using dynamic contrast-enhanced MRI to measure the BBB leakage rate of Gd-DOTA (K^trans^ [min^-1^]), a biomarker for BBB integrity, revealed an identical biphasic entry pattern post-ICH to that seen with Dil-Lp (Figure 4B-C). The mean Gd-DOTA leakage rate (*K*^trans^) tended to increase between 48-72 h, but was not statistically significant. To investigate further, we assessed only the areas of greatest BBB disruption, calculating the 95 % percentile of lesion *K*^trans^ values (Figure 4D). In this analysis, leakage was significantly higher at 72 h indicating that while the average BBB integrity may be similar between 48-72 h, at 72 h certain focal areas of the lesion exhibit hyper-permeability. These observations were further confirmed in a larger cohort of mice (n=9-10) by measuring *K*^trans^ values at 24 h and 72 h post-ICH (Figure 4E) which showed a significant increase in mean *K*^trans^ values confirming the second phase of BBB disruption. Given the similarity in the temporal and spatial pattern of liposome and Gd-DOTA accumulation in the brain, it is likely that their penetrance is governed by a common entry process mediated by BBB disruption. These findings strongly suggest that leakage of both molecules into the brain are mediated by self-diffusion across the disrupted BBB.

### Both transcellular transport and tight junction disassembly contribute to the entry of liposomes into the brain after ICH

To investigate the mechanism of liposomal entry into the brain, IHC staining for Caveolin-1 (Cav1) and Claudin-5 was performed to evaluate the contribution of transcytosis transport and tight junction (TJ) disassembly respectively. A significant increase in Cav1 expression 3-5 h after ICH which suggests that enhancement in transcytosis transport is contributing to liposomal brain accumulation at this time. This agrees with our previous work in ischemic stroke that showed enhanced Cav1 expression between 0.5-2.5 h after the middle cerebral artery occlusion (MCAo) model of stroke. In addition, we stained for Claudin-5 to evaluate TJ remodelling after ICH, a significant reduction in Claudin-5 being seen at 3-5 h and 48-50 h, which suggests a contribution of paracellular transport to liposomal accumulation at both time points. Overall, our data (Figure S4-5) suggest liposomal entry into the brain in ICH is mediated by; 1) an early phase of enhanced transcellular transport and TJ disassembly, and (2), a delayed phase (48 h after ICH) of TJ disassembly.

### ICH lesions exhibited increased but stable *T*_1_ compared to naïve mice

Elevated spin-lattice relaxation time (*T*_1_) was observed in the ICH lesions (white columns) compared to normal tissue in the same location in sham mice (grey shaded columns) indicating the presence of oedema (likely vasogenic) (Figure 4F). No significant changes in *T*_1_ values were observed over the time frame tested in our experiment (up to 72 h) which suggested the persistence of brain oedema past 72 h. Analysis of T2-TurboRARE images for the changes in ipsilateral/contralateral ventricle size ratio in ICH mice showed no significant differences at this stage of the disease (Figure 4 G-H).

### ICH lesions exhibit increases in free iron content, T_2_*-weighed MRI signal intensity and decreases in RBCs over time

Accumulation of iron products in the brain was assessed by gradient-echo MRI (*T*_2_* signal). At 3 h post-ICH, a significant reduction in *T*_2_* signal intensity was observed in ICH mice (white columns) compared to sham mice (grey columns), likely reflecting penetrance of iron-rich RBCs into brain tissue from the initial bleed. By 48 h, T_2_* signal intensity returned to the level observed in healthy tissue of naïve mice, indicating dispersion of RBC iron into the lesion due to RBC lysis.

**Figure 5:**
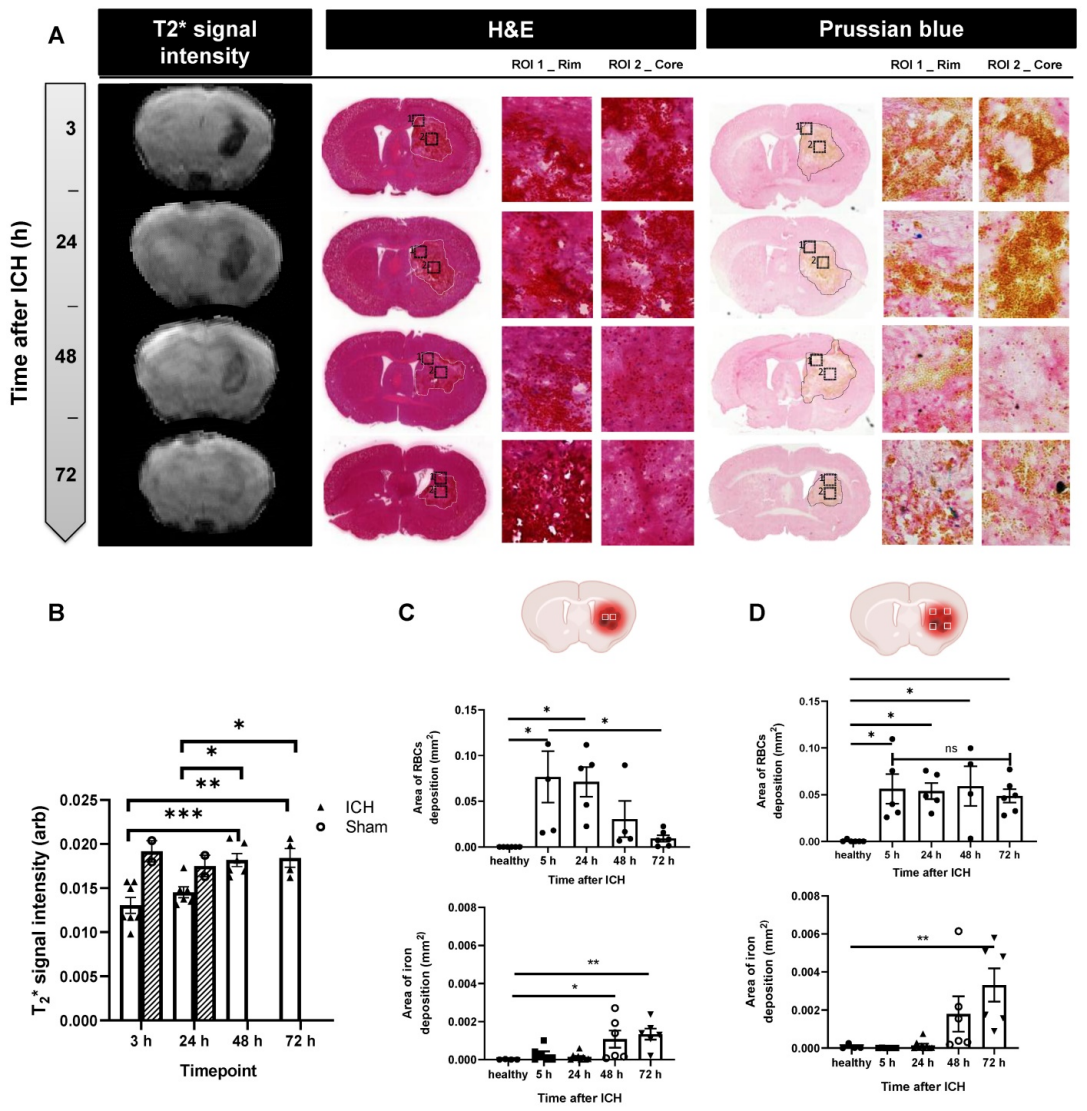
A) Accumulation of iron in the brain was assessed by T2* signal intensity, H&E and Prussian Blue staining. B) Our data showed a sharp reduction in T2* signal intensity at 3 h, indicative of deposits of blood-products (heme iron) within the lesion (white columns) compared to sham mice (grey columns), likely reflecting penetrance of iron-rich RBCs into brain tissue from the initial bleed. By 48 h, T2* signal intensity increased back to the level observed in the healthy tissue of naïve mice, indicating clearance of blood products from the brain. These results were confirmed by histology using H&E and Prussian Blue stain in the lesion core (C) and lesion rim (D). At each brain section measurements of positive RBCs, areas and positive free iron stain were done on four rim regions and two core regions. H&E images indicated RBCs positive area decreased over time in the lesion core while the values are not significantly changed in the lesion rim. Prussian Blue stain on the other hand indicated a continuous rise in free iron as a result of RBCs haemolysis reaching significant levels around 72 h after ICH. Values are expressed as mean ± SEM. Statistical analysis was performed using one-way analysis of variance followed by the Tukey multiple comparison test and p values < 0.05 were considered significant.

The spatial distribution of T_2_* changes covered the entire lesion at 3 h and progressed to affect only the perihematomal rim at 48-72 h (Figure 5A-B). This may reflect either i) preferential clearing of RBCs from the lesion centre leaving RBCs in the rim unaffected, ii) migration of RBCs to the peri-haematoma rim, or iii) accumulation of free-iron in the peri-haematoma rim to levels that cause detectable T_2_* effects.

To understand contributions to T_2_* signal intensity, histological analysis of RBCs and free iron deposition based on H&E and Prussian Blue stain was performed at three different coordinates from the Bregma; +0.14 (Figure 5C-D), -0.58 (Figure S6 A-C) and -1.22 (Figure S6 B-D) both at the lesion core and perihematomal area (rim). Analysis of RBCs positive areas with H&E confirmed a significant decrease in RBCs in the core over time, correlating with the progressive increase in T_2_* signal intensity. Quantification of RBCs accumulation in the brain assessed via H&E staining did not show significant change over time in the lesion rim (Figure 5D). Conversely, free iron as measured by histology using Prussian Blue stain increased with time in both the core and rim (Figure 5 C-D and Figure S6), supporting the progressive release of iron from RBCs as a result of haemolysis, and possible migration of free iron from the centre to the edge of the lesion. Lack of correlation of free-iron changes in the core with T_2_* MRI likely reflects the dominant effect of RBC (bound iron) towards T_2_* signal loss. At 72 h, T_2_* signal loss was prevalent only in the rim where free iron was also found to be high. These findings may reflect highly concentrated free iron or potential rebleeding in this area. These changes in iron content and haematoma resolution could have affected liposomal transport after leakage into the brain and may explain some variation in liposomal brain accumulation observed between timepoints i.e the changes in redistribution of liposomes into the core in the second phase 48-72 h.

### Microglial activation is a dynamic process following ICH and co-localised with DiI-liposomal accumulation

Microglia and astrocytes play a key role in ICH pathology, thus we next investigated if liposomes co-localised with those cells after access into the brain. Confocal images showed that microglia are ramified illustrating their transition into reactive, phagocytic and amoeboid cells which were observed both at the lesion core and periphery (Figure S7). Astrocytes, identified as glial fibrillary acidic protein (GFAP) +ve area, were observed to be most evidently activated at the peripheries of the lesion, indicating reactive gliosis. Similar to our previous experimental design, DiI-Lp were injected at three different time points after ICH (3, 24 and 48 h) and representative confocal images were taken 24 h after DiI-Lp injection. Quantification of DiI-Lp co-localisations with Iba1 +ve cells (activated microglial marker) indicated that maximum uptake of DiI-Lp by microglia was observed when injected at 3 h (44 %) and 48 h (32 %) after ICH, which was significantly higher than that observed when injected 24 h post-ICH (9 %). These data correlate with biphasic liposomal uptake into ICH lesions and suggested two windows for optimum microglial targeting to shift or direct their activation. Unlike uptake by microglia, only limited uptake of DiI-Lp was detected in astrocytes at all time points (Figure S7 and S8).

## Discussion

Recently we have demonstrated in an experimental ischaemic stroke model that liposomes can infiltrate BBB at the ischaemic site taking advantage of enhanced transcytosis and tight junction disassembly 20. Liposomes also have great potential for targeted drug delivery to damaged tissue following ICH. Here we aimed to confirm the penetrance of liposomes in ICH, possible underlying mechanisms by which liposomes enter the brain, and to evaluate the optimal timepoint for drug delivery.

We have demonstrated that liposomes accumulate in the lesion site after ICH and maintain long term co-localisation in the injured area. A biphasic entry pattern was identified, indicating 3 h and 48 h post-ICH as the optimum windows for therapeutic intervention. We also observed that liposomal distribution in the brain varies over time and is likely linked to haematoma resolution. Activated microglia uptake the liposomes at both early and late time points which could be related to enhanced phagocytic capacity after ICH. By this, we have demonstrated the ability of a clinically approved nanomedicine formulation to cross the BBB in a rodent model of ICH, providing promising data for future therapeutic studies.

Our data showed that the biphasic entry pattern of the liposomes into the brain correlates with leakage of Gd-DOTA, as detected using DCE-MR. These observations are very similar to our previous findings in ischaemic stroke which also showed two windows of liposomal entry into the brain that peaks around 3 h and 48 h after brain ischaemia 20. Although these observations suggested that there is an overlap in the timing of BBB disruption between the two conditions, the underlying pathological mechanism is different. In ischaemic stroke, the lack of oxygen and glucose supply to the brain initiate the acute stage of BBB disruption whereas delayed BBB damage is associated with reperfusion injury and infiltration of inflammatory cells to the brain 19. In ICH, the leakage of blood components into the brain activates pathways related to cell injury and inflammation which has a major role in BBB disruption. The role of cerebral ischaemia in ICH injury is not yet conclusive. Perihematomal blood flow measurements in animal and human studies are not significantly low to trigger ischaemic brain injury 30-32. The differences in the mechanism of BBB disruption between the two models may also explain the higher extent of liposomal entry into the brain we observed here in ICH, which is 10 times higher than what we reported in ischaemic stroke 20, offering the potential for enhanced drug delivery in this, particularly devastating stroke subtype.

The onset of BBB disruption in ICH reported in previous studies is rather variable depending on the animal model. Induction of ICH by injecting blood into the brain showed no early BBB disruption at 4 h, but BBB damage was observed at 1-2 days 33. Inducing ICH by collagenase injection resulted in BBB disruption within 30 minutes followed by a prolonged, but lower level of BBB disruption that was shown to last for 7 days 18 which agrees with the biphasic BBB disruption we observed. Another study with the collagenase ICH model showed a continuous increase in BBB disruption over time that peaks around 3-4 days before returning to normal level at 7-10 days 34. However, that study did not show BBB permeability measurements in the first few hours after ICH which could explain the absence of biphasic BBB disruption. In humans, ICH is reported to cause both early 35 and delayed vascular permeability 36 and, in some patients, continuous bleeding and extravasation of contrast agent were observed in the first 24 h post-injury 35,37. Therefore, the findings in this study confirm that the collagenase model recapitulates the clinical picture of BBB disruption. The detection of endogenous IgG infiltration into the lesion site is frequently used to evaluate BBB disruption as indicated in our findings. While this method is adequate in concluding the presence of BBB damage, it is not possible to determine the exact timings at which the damage occurred. Therefore, in this study, it was essential to correlate our observations with serial in-vivo MRI to determine the time-course of BBB disruption post-ICH. In our previous study, investigating the mechanism of liposomal entry into the brain after ischaemic stroke, we demonstrated that liposomes access to the brain is mediated by enhanced caveolae-mediated transcytosis in the acute phase (0.5-2.5 h after stroke) when no damage to the TJ is detected 20. In this study, we observed that Cav1 is also significantly enhanced 3-5 h after ICH which suggests that liposomes transport across a disrupted BBB is linked to caveolae-mediated transport. Our data also suggest the contribution of TJ remodelling to liposomal entry into the brain both at 3-5 h and 48-50 h after ICH as indicated by the reduction in claudin-5 positive areas. Previous studies in rodent models of ICH reported a reduction in various TJ proteins 1-3 days after ICH 38-41 using immunohistochemistry, western blot or mRNA expression. Similarly, a clinical study in ICH patients showed elevated TJ protein levels in CSF samples measured on admission which indicate a role in TJ remodelling in BBB dysfunction very early after ICH 42.

In the present study, we assessed BBB disruption and liposomal entry into the brain up to 72 h post-ICH, however, other studies in animals 34,43 and human 36 reported that BBB disruption lasts over a longer period before reaching baseline level. This suggests that liposomal entry into the brain could be extended beyond the 72 h tested in this study. Further research will be needed to confirm the exact mechanism of entry and whether this would change over time given the variation in the underlying pathological events associated with ICH.

In this study, we have used serial measurements of spin-lattice relaxation time (*T*_1_) to evaluate brain water content (oedema) after ICH 44. Our data indicated elevated levels of oedema detected in the ICH model compared to naive mice. Using T2-weighted MRI and apparent diffusion coefficient (ADC) measurement Yang et al showed an increase in perihematomal oedema after ICH reaching a peak level at day 3 followed by a gradual decrease over 7 days 34. However, no earlier measurements were performed in that study. In agreement with our findings that study also showed diffuse microglial activation that covers both the lesion core and periphery, while astrocytes activation was observed in the perihematomal area. Free iron deposition was also observed mainly in the lesion rim at day 3 indicating the start of RBCs lysis. These data collectively suggested that activated glial cells and RBCs lysis at the lesion periphery may play a role in increasing BBB permeability and vasogenic oedema in this region.

The rupture of the microvascular wall in ICH exposes neurons, astrocytes, microglia and other cells around the haemorrhagic area to blood-derived iron, either bound or free. Erythrocyte lysis is reported to start within minutes after bleeding and lasts for days afterwards 45. Our data showed a sharp reduction in T_2_* signal intensity at 3 h, indicative of deposits of blood-products (heme-iron) within the lesion. This appears to resolve by 48 and 72 h post-ICH, with T_2_* signal returning to normal first in the core, and last in the lesion rim. This suggests that the second phase of BBB hyper-permeability at 72 h does not result in significant re-bleeding of RBCs into the lesion. Free iron was found to accumulate over time, likely the result of RBC lysis. Taken together, these findings suggest that in the context of ICH, T_2_* signals are dominated by iron within RBCs. We hypothesize that as these cells rupture and release iron into the surrounding area, the iron disperses resulting in a reduction in susceptibility gradients and an increase in T_2_*. Much higher levels of free-iron were observed in the lesion rim (2-3 fold) compared to the lesion core. This indicates that the rim may also contain a higher density of RBCs (the source of free iron), or is a site of significant free iron accumulation, which is supported by Figure 5D and Figure S6 C and D. Iron and heme-iron lead to the induction of reactive oxygen species which can lead to an iron-dependent cell death known as ferroptosis 46,47. Although the concept is still evolving, ferroptosis is now considered a unique form of regulated cell death characterized by the cytosolic accumulation of iron and executed by the massive induction of lipid ROS that leads to intracellular oxidative stress and damage to nucleic acid, proteins, lipid membrane and consequently cell death 48.

Previous studies in animal models of ICH showed that efficient haematoma clearance is usually a delayed process that starts 3 days post ICH which correlates with the peak of microglial activation and is completely resolved around 3-4 weeks 34,49,50. This agrees with our data that showed no significant reduction in RBCs deposition area, haematoma volume and infarct volume up to 72 h post-ICH.

Microglia are activated within minutes after ICH onset and play a key neuroprotective role by clearing blood products, apoptotic cells and cellular debris through phagocytosis 50,51. This process has an important role in providing a nurturing environment to promote tissue recovery, however excessive microglial activation and infiltration of peripheral inflammatory cells can also exacerbate ICH injury through the release of proinflammatory mediators 50. CD36, a member of the class B scavenger receptor family, is an important mediator of phagocytosis and it is highly expressed in microglia and mediates the phagocytosis of apoptotic cells, bacteria and low-density lipoproteins 52,53. Previous studies suggested that CD36 has a critical role in controlling microglial phagocytic activity. The expression of CD36 in microglia is transcriptional regulated via the activation of peroxisome proliferator-activated receptor (PPAR) which accelerates their RBCs phagocytic capacity and haematoma resolution 54. Treatment with PPAR agonist (e.g. rosiglitazone) showed enhanced RBCs clearance by increasing CD36 expression and phagocytosis both in vitro and in vivo and in turn promoting hematoma resolution 10. PPAR activator also modulates the proinflammatory mediator expression to reduce secondary brain damage and protect other brain cells from oxidative damage imposed by microglia during phagocytosis. Previous studies showed that Toll-like receptor (TLR) may play a role in regulating CD36 receptor function both in phagocytosis and inflammation. Upregulation of CD36 expression was demonstrated in TLR-4 knockout mice suggested that TLR-4 suppression could promote hematoma resolution 49,55. The enhanced uptake of liposomes observed in microglia compared to astrocytes could be linked to activated phagocytosis in microglia due to their critical role in RBCs clearance and haematoma resolution.

Since microglia are activated as early as 1 h after ICH and remain activated for the first few days, the difference in liposomal uptake after ICH reflects the differences in liposomal brain entry levels showing a dual uptake pattern. Given the biphasic therapeutic windows observed with liposomes, a similar dual therapeutic approach can be designed to enhance haematoma clearance and minimise the deleterious proinflammatory responses that could trigger further brain damage.

The critical role of activated microglia in haematoma resolution and their localisation both in the lesion core and periphery may also explain the difference in liposomal localisation in the brain parenchyma observed 48-72 h after ICH. Activation of MMPs that are involved in extracellular matrix remodelling is also peaked 24-72 h after ICH 51,56. Their critical role in delayed BBB disruption and neurovascular remodelling suggested that they could be linked with the alterations in liposomal distribution over time 57-59.

The benefits of nanomedicine-based drug delivery in stroke research have been demonstrated recently, however, these have been largely restricted to ischaemic stroke models 20,60,61. No liposomal formulation has been tested before in ICH, but very limited studies with other types of nanoparticles showed promising results in haemorrhagic stroke. One study tested the therapeutic effect of nanoemulsion formulation of quercetin in a collagenase rat model and demonstrated superior antioxidant effect, reduce haematoma volume and improved functional recovery compared to the free drug 62. Another approach using polylactic-co-glycolic acid nanoparticles of human recombinant erythropoietin decreased the rate of lethal events and improved neurological outcomes when tested in a traumatic intracerebral haemorrhage model 63.

Other strategies that have been widely investigated to enhance brain prenetration include active targeting. In this case, a high-affinity ligand against an influx system or highly expressed receptors on the brain endothelial cells is utilised to shuttle molecules across the BBB irrespective of the alterations of BBB integrity. Transferrin receptor (TfR), insulin receptor and glutathione (GSH) transporter are some examples of receptors present on the BBB that have been utilised to enhance drug brain penetration. Using this strategy in mice to deliver erythropoietin 64 or type II tumour necrosis factor-α by fusion with TfR monoclonal antibody resulted in high brain accumulation reaching 2-3 % of ID / g tissue 65. This enhanced brain penetration is in the same range observed in our ICH model where we demonstrated 2-3 % of ID / g brain tissue of liposomal accumulation 3-24 h after ICH. This is also comparable to what was reported before from highly lipophilic molecules such as diazepam reaching 1.8% of ID / g brain tissue within 10 minutes of I.V injection 66. In addition Targeting receptors at the BBB improves the brain penetration of immunoliposomes and their encapsulated drug into the brain. From those TfR and GSH targeted immunoliposomes have been widely investigated for brain tumours 67,68, and multiple sclerosis 69,70. Using immunoliposomes delivery method increased drug accumulation in the brain by 3-4 folds was observed compared to the free drug reaching up to 0.06 % of ID / g tissue in some examples 71.

An exciting observation in this study when considering clinical translation is the extended therapeutic window provided by the significant liposomal accumulation when injected in the acute phase (3 h post-ICH) and the subacute phase (48 h post-ICH). The broad treatment timing provided by liposomes means a larger subset of patients can benefit and the choice of therapeutic molecules could be diverse based on the underlying pathological mechanisms dominating at that time. The development of liposomal drug delivery systems is versatile in terms of treatment choice and has already demonstrated promising results in preclinical models of ischaemic stroke models 20,60,61. The safety profile of liposomes is already established through its current licenced use in other pathologies 72 and thus holds the best chances of being fast-tracked to the clinic. Modifying liposomes to scavenge iron or modulating microglial activity towards enhanced haematoma resolution may enable therapies the will enable the reduction of these pathologies, the effect of which could be tracked using MRI. Moreover, this study will provide a potential direction for the development of liposome-based imaging contrasts for the ICH model in the future in line with other clinically relevant fluorescent imaging applications 73-75.

## Conclusion

This study provides evidence that liposomes can selectively accumulate within the lesion site in a collagenase model of ICH. Liposomal entry into the brain peaked when injected 3 h and 48 h after ICH and correlated with the changes in leakage of Gd-DOTA into the lesion detected using DCE-MRI, indicating the primary mechanism of penetrance was via self-diffusion across the disrupted BBB. Liposomal accumulation observed is very specific to the haemorrhage area and showed 40 % co-localisation with activated microglia at the lesion site. These observations offer an extremely promising technology for ICH treatment enabling time-specific new therapies and spur the rejuvenation of drugs that have been tested before in ICH but have been missed due to failed efficacy or unwanted peripheral toxicity.

## Materials and Methods

### Materials

The lipids used in this study including hydrogenated soy phosphatidylcholine (HSPC) and 2-distearoyl-*sn*-glycero-3-phosphoethanolamine-*N*-[methoxy(polyethylene glycol)-2000] (DSPE-PEG_2000_) were a kind gift from Lipoid GmbH (Ludwigshafen, Germany). 18:0 PE-DTPA 1,2-distearoyl-*sn*-glycero-3-phosphoethanolamine-N-diethylenetriaminepentaacetic acid (ammonium salt) was from Avanti Polar Lipids (USA). Chloroform and methanol were purchased from Fisher Scientific. Phosphate buffer saline, cholesterol, paraformaldehyde, sucrose, goat serum, collagenase and cresyl violet were purchased from Sigma. Polycarbonate extrusion filters (Whatman) 800nm, 200nm, and 100nm were from VWR, UK. PD-10 desalting columns were bought from GE-Healthcare Life Sciences. 1,1'-Dioctadecyl-3,3,3',3'-Tetramethylindocarbocyanine Perchlorate (DiI) was purchased from Invitrogen Detection Technologies. Biotinylated anti-mouse IgG, NHS blocking solution, DAB peroxidase (HRP) substrate kit and Vectastain Elite ABC HRP Kit were all purchased from Vector Laboratories.

### Mice and diets

We have used C57BL/6 male mice (11-12 week-old, weighing 25-30 g) provided by Charles River, United Kingdom. The mice were housed in groups of 4-5 with free access to diet and water and at a constant ambient temperature of 21 ± 2 °C and humidity of 40-50 %, on a 12 h light, 12 h dark cycle. Experiments involving animals were all performed according to the United Kingdom Animals (Scientific Procedures) Act, 1986 (P28AA2253) and the work is already approved by the Home Office and the local Animal Ethical Review Group, University of Manchester and reported in compliance with the ARRIVE guidelines. Most of the studies conducted included 4-5 animals per group and analysis was performed blinded to the treatment conditions.

### Induction of Intracerebral haemorrhage (ICH)

Before induction of ICH, mice were acclimatised to mash and had their bodyweight assessed. To induce ICH, mice were anaesthetised with a mixture of 4% isoflurane in a mixture of 30 % oxygen and 70 % nitrous oxide. to shave the region between eyes and ears, before being transferred to a feedback-controlled heating pad where the anaesthesia was maintained at 1.5-2 % of isoflurane using a nose cone. A longitudinal midline incision through the skin was made and soft tissue retracted. During the surgical procedure, core body temperature was monitored using a rectal probe and maintained at 37 ± 0.5 °C, using a thermal blanket. A burr-hole was created at the following coordinates; bregma 0, lateral -2.0 and dorsoventral -2.5 using an electric hand drill. A glass micropipette was used to inject 0.03 U collagenase VII S, dissolved in 0.5 µl sterile saline, at a rate of 1 µl / min. The needle was left in place for 10 minutes before a gradual withdrawal over a period of 60 seconds. The wound was then closed with nonabsorbable sutures. Before recovery, all mice were given saline (1 ml / 100 g body weight) and buprenorphine (0.05 mg / kg S.C). Following the surgery, the mice were then housed in a cage, with a choice of both mash and pelleted food, in a heated cabinet (~25 °C). Mice were monitored until they had recovered from the anaesthetic and were completely conscious and re-gained their righting reflex. After surgery, mice were weighed every day and assessed for their general well-being. Bodyweight data were presented as a % weight change compared with bodyweight on the day of surgery. Assessment of neurological function following ICH was performed with the 28-point neurological scoring system 20.

### Assessment of brain damage with cresyl violet staining

Brain sections were stained with cresyl violet and the infarct volume was calculated by measuring the areas of neuronal loss at eight defined coronal levels as previously described 20. On each section, the area of damage was measured using ImageJ (NIH, Bethesda, MD, USA), adjusted for oedema and the volume of damage was calculated by integrating of areas of damage with the distance between coronal levels using GraphPad Prism 7, Software. The volume of damage was expressed as the total amount of ischaemic damage.

### Haematoxylin and Eosin staining

For the assessment of haemorrhagic regions, haematoxylin and eosin (H&E) staining was performed. Frozen sections 20 µm thick were first fixed in formal alcohol for 20 seconds and rinsed quickly in distilled water. Following that we stained in haematoxylin for 2 minutes and washed in Scott's tap water. The slides were then stained in eosin for 10 seconds, and rinsed in tap water, before being gradually dehydrated in alcohol (70-100 %) in three steps. Once the slides are dehydrated, they were cleaned twice in Xylene each for 1 minute before being cover slipped using a resinous mounting medium. Slides were scanned using a slide scanner (3D Histec Pannoramic 250) and images were captured using Pannoramic Viewer. The area of red blood cells was measured in the same way as the infarct volume was calculated and compared between the groups.

### Preparation of DiI-labelled liposomes (DiI-Lp)

DiI-labelled liposomes composed of HSPC:Chol: DSPE-PEG_2000_ 56.3:38.2:5.5 mol/mol % were prepared by thin-film hydration method followed by extrusion 20. Briefly, lipids dissolved in chloroform: methanol mixture (4:1) were mixed in a round bottom flask and 5mol% of DiI in ethanol (1 mg / ml) was added to the lipid mixture. Organic solvents were then evaporated to produce the lipid film. Lipid films were kept protected from light and hydration was performed with HBS (20 mM HEPES, 150 mM NaCl, pH 7.4) to a final lipid concentration of 12.5 mM. To produce small unilamellar liposomes, the size was reduced by extrusion through 800 nm and 200 nm polycarbonate filters 5 times each then 20-40 times through 100 nm membranes using a mini-Extruder (Avanti Polar Lipids, Alabaster, AL).

### Preparation and characterisation of ^111^In-labelled liposomes (^111^In-Lp)

To evaluate the accumulation of liposomes in the brain after ICH, we have used real-time SPECT/CT imaging and gamma counting. Liposomes were radiolabelled with radioactive indium (^111^In). Briefly, 25 mM (total lipid concentration) of HSPC:Chol:DSPE-PEG_2000_:PE-DTPA 56.3:38.2:5.5:1 mol/mol % liposomes were prepared as described above using the thin-film hydration method. Hydration of the lipid film was done with freshly prepared ammonium acetate buffer (0.095 M, pH 5.5) at 60°C followed by extrusion to reduce the size of the liposomes. Subsequently, liposomes were radiolabelled by 1 h incubation with radioactive **^111^**InCl_3_ (11 MBq / 2.5 µmol lipids) in 0.2 M ammonium acetate pH 5.5. Incubation was carried out at room temperature with continuous vortexing every 5 minutes. At the end of incubation 0.1 M, EDTA (1/20 of the total volume) was added to chelate any free ^111^In. To determine the radiolabelling efficiency, any unbound ^111^In and ^111^In-EDTA were removed with PD-10 column pre-equilibrated with HBS pH 7.4. Aliquots of each final product were diluted five folds in PBS and then 1 μl was spotted on silica gel impregnated glass fibre sheets (PALL Life Sciences, UK). The strips were developed with a mobile phase of 50 mM EDTA in 0.1 M ammonium acetate and allowed to dry before analysis. This was then developed and the autoradioactivity was quantitatively counted using a cyclone phosphor detector (Packard Biosciences, UK). The immobile spot on the TLC strips indicated the percentage of radiolabelled ^111^In-Lp, while free ^111^In was detected as the mobile spots near the solvent front. Very minimum free ^111^In was detected to yield radiolabelling efficiency of > 85 % as displayed in Figure S1. The radiolabelling stabilities of the final product of ^111^In-Lp were studied after five times dilution in both 50 % serum or PBS and then incubated at 37 °C up to 48 h. At different time points (0, 1 and 24 h), 1 μl of the aliquots was spotted on silica gel impregnated glass fibre sheets and then developed and quantified as described above.

### Liposomes characterization

Liposome size and surface charge were measured using Zetasizer Nano ZS (Malvern, Instruments, UK). Samples were diluted 100 times with purified distilled water before measurements. Triplicates measurements were recorded, and the data were expressed as average ± S.D. The fluorescence intensity of DiI-Lp was recorded using Carry Eclipse fluorescence spectrophotometer, (Agilent Technology). Samples were first diluted 200 times in HBS and recorded at 518 nm / 565 nm excitation/emission wavelengths (slit 5 / 10).

### Assessment of DiI-Lp accumulation into the brain with optical imaging

To confirm the accumulation of the liposomes in the brain, ex vivo optical imaging was used by detecting the optical fluorescence signal of the liposomes. IVIS imaging was performed shortly after cardiac perfusion with ice-cold 0.9 % saline followed by 4 % PFA to remove any DiI-Lp that are still circulating in the blood. DiI-Lp were administered at the following time point after ICH (3 h, 24 h and 48 h) by I.V and the brain tissues were extracted either 2 h or 24 h after I.V administration. Imaging was performed with the IVIS Lumina II imaging system (Caliper Life Sciences Corp., Alameda, CA) at 535 nm / DsRed excitation and emission filters with 0.5 sec exposure. DiI-Lp total fluorescence intensity in the brain was quantified by drawing a region of interest (ROI) that covers the whole brain and is expressed as total efficiency.

### Single-photon emission computed tomography (SPECT/CT)

Mice were subjected to anaesthesia *via* the inhalation of 2.5 % isoflurane in a mixture of 30 % oxygen and 70 % nitrous oxide. Each animal was then intravenously injected with 200 ul of the radioactive ^111^In-Lp (8-9 MBq). At different time points after injection (t= 0-1 h, & 24 h) SPECT/CT imaging was carried out using a Nano-Scan^®^ SPECT/CT scanner (Mediso, Hungary). SPECT images were obtained in 20 projections over 40-60 min using a 4-head scanner with 1.4 mm pinhole collimators. CT scans were taken at the end of each SPECT acquisition using a semi-circular method with a full scan, 480 projections, maximum FOV, 35 kV energy, 300 ms exposure time and 1-4 binning. Acquisitions were done using the Nucline v2.01 (Build 020.0000) software (Mediso, Hungary), while the reconstruction of all images and fusion of SPECT with CT images was performed using the Interview™ FUSION bulletin software (Mediso, Hungary). The images were further analysed using VivoQuant 3.0 software (Boston, US) where the SPECT images with scale bars in MBq were corrected for decay and the slight differences in radioactivity in the injected doses between animals. For a quantitative assessment of ^111^In-Lp in the brain, a cut and count method was used. Mice were anaesthetized by isofluorane inhalation and each mouse was injected *via* the tail vein with 200 μl containing ^111^In-Lp labelled with approximately 8-9 MBq. 24 h after injection, mice were perfused with iced cold saline (0.9 %) followed by PFA (4 %) to remove any ^111^In-Lp from the blood before brain tissues were collected. Each sample was weighed and counted on a gamma counter (Perkin Elmer, USA), together with a serial dilution of the injected dose. The results were represented as the percentage of the injected dose (% ID / gm tissue ± SEM), n = 4-5 mice per group.

### Assessment of BBB permeability to IgG

To assess BBB permeability, we have stained for endogenous IgG infiltration into the brain was visualised using peroxidase-based immunohistochemistry. Serial brain sections (20 µm thick) were mounted on slides and washed 3 times with PBS. Following that endogenous peroxidase activity and non-specific staining were blocked by 10 min incubation in 0.3 % H2O2 and then washed (3 times in PBS, 10 min each). blocking was performed with 10 % normal horse serum (NHS) in 0.3 % Triton X-100 PBS (PBST) for 1h before overnight incubation with biotinylated anti-mouse IgG (1:250 in 0.3 % PBST, Vector Laboratories) at 4°C. Sections were then incubated with avidin-biotin-peroxidase complex, and colour-developed using freshly prepared diaminobenzidine (DAB) solution. To ensure comparable DAB staining between sections is achieved, the time of the colour change was recorded, and DAB was applied for each subsequent sample for the same amount of time.

### Immunohistochemistry staining for Cav1 and CD31

Free-floating serial brain sections (20 µm thick) were washed 3 times in PBS for 10 min and blocked for 1 h in 10 % normal goat serum (NGS) in 0.3 % PBS triton (PBST). This was followed by overnight incubation with primary antibodies in 2 % NGS in PBST at 4 °C. The primary antibodies used are; rat anti-mouse CD31 (BD Pharmingen, 550274, 1:100) and mouse anti-mouse Cav1 (BD Biosciences, 610407, 1:50). After incubation with the primary antibodies, sections were washed 3 times in PBS and incubated with fluorescently labelled secondary antibodies in 2 % NGS in PBST. To visualise the primary antibodies, the following secondary antibodies were used; goat anti-mouse Alexa Fluor® 488 conjugate (Invitrogen A11001, 1:500), goat anti-Rat Alexa Fluor® 647 conjugate (Invitrogen, A-21247, 1:200). In the end, samples were washed 3 times in PBS and transferred on non-gelatine coated slides and left to dry overnight before slides were then coverslip with ProLong Gold Antifade Mountant (Thermo Fischer Scientific, Inc., USA). Images were collected on either SP5 inverted microscope using 40x objective.

Brain sections injected with DiI-Lp at 3 h and 48 h were used for this study and analysed 24 h after liposomal administration. The ipsilateral ICH lesion site and the corresponding areas on the contralateral slide were analysed. For each brain region, 4 discrete areas were imaged at a magnification of 40 x. Each area was analysed in ImageJ by applying fluorescence intensity threshold and the number of pixels from each colour (black, red, green, yellow, blue, magenta) were then automatically measured as previously described 76. These values enabled us to ascertain the area of immunolabeling from each cell type and their co-localisation with the liposomes. Data expressed as average ±STD.

To complement confocal images, widefield overview images were obtained at x40 magnification without oil utilising Leica Thunder imager microscope. All tissue areas were processed under identical conditions to demonstrate liposomes (red), CD31 (blue) and caveolin-1 (green) immunoreactivity. After initial imaging concluded, mosaic merge processing via Leica application suite X v3.7.4.23463 combined tiles into one image and large volume computational clearing, such as lightning and thunder programme, reduced out of focus areas to produce high quality images.

### Immunohistochemistry staining for claudin-5

Serial frozen sections of brain tissues (20 µm in thickness) were cut on a cryostat, mounted on slides and allowed to warm to RT for 20 minutes before being rehydrated in PBS for 10 min. Following that the slides were quickly washed in tap water and left to dry vertically at 37 ˚C for 1 h. The slides were then blocked for 1 h at room temperature in 5 % goat serum solution prepared in 1 % BSA, 0.1 % Triton X-100 (as penetration enhancer) and 0.05 % Tween 20 (used detergent and surface tension reducer). This was followed by overnight incubation with rabbit anti-mouse Claudin-5 in 2 % Donkey serum in 1% Bovine serum albumin, 0.05 % Tween 20, 0.3 % Triton X-100, 1:100 (Abcam, Cambridge). After incubation with the primary antibodies sections were washed 3 times in 0.1 % tween solution PBS and incubated with donkey anti-rabbit Alex 488, 0.1 % Tween 20 1x PBS, 1:200 (Invitrogen, USA). In the end, samples were washed 3 times in 0.1 % PBS tween and left to dry overnight before slides were then coverslip with ProLong Gold Antifade Mountant with DAPI (Thermo Fischer Scientific, Inc., USA). Images were collected on an inverted SP5 confocal microscope using 63x objective in the striatum. Analysis of the images was performed in the same way described above for Cav1 staining.

### Immunohistochemistry staining for astrocytes and microglia

Serial frozen sections of brain tissues (20 µm in thickness) were cut on a cryostat, mounted on slides and allowed to warm to RT for 20 minutes before being rehydrated in PBS for 10 min. Following that the slides were quickly washed in tap water and left to dry vertically at 37 ˚C for 1 h. For Iba-1 staining only, antigen retrieval was first performed. For this, the slides were placed inside a closed glass Coplin jar filled with the EDTA solution (pH 6.0). The jar was sealed with parafilm and placed inside a 500 ml glass beaker filled with boiling water and placed on a hot plate for 10 min. After boiling the slides were immediately rinsed with ultrapure Type I water at room temperature for 5 min followed by two successive rinses in ultrapure Type I water for an additional 5 min. The slides were then blocked for 1 h at room temperature in 5 % goat serum solution prepared in 1 % BSA, 0.1 % Triton X-100 (as penetration enhancer) and 0.05 % Tween 20 (used detergent and surface tension reducer). This was followed by overnight incubation with primary antibodies in 2 % normal goat serum, 1 % BSA and 0.3 % Triton X-100 in PBST at 4 °C. The primary antibodies used in the study are explained in detail below; Chicken antiGFAP (Abcam AB4674, 1:200), Rabbit antiIb1a (Wako 019-19741, 1:100). After incubation with the primary antibodies sections were washed 3 times in 0.1 % tween solution PBS and incubated with fluorescently labelled secondary antibodies prepared in 0.1 % PBS tween solution. To visualise the primary antibodies, the following secondary antibodies were used; goat anti-Ck Alexa Fluor® 488 conjugate (Invitrogen A11039, 1:500), goat anti-Rabbit Alexa Fluor® 647 conjugate (Invitrogen, A21244 1:200). In the end, samples were washed 3 times in 0.1 % PBS tween and left to dry overnight before slides were then coverslip with ProLong Gold Antifade Mountant with DAPI (Thermo Fischer Scientific, Inc., USA). Images were collected on an inverted microscope using either 40x or 63x objective in the striatum and the outer cortex at bregma -0.58 mm.

Analysis of liposomal uptake in microglia and astrocytes was done by Imaris software based on Z-stack confocal images reconstructed into 3D images using a method previously described 77,78. Using Imaris software a mask is first created over each channel corresponding to liposomes, microglia and astrocytes. Figure S7 showing a representative images of liposomes (red) colocalised mainly with microglia (cyan), while limited interaction is observed with astrocytes (green). All tissue samples are processed using the same acquisition settings. The co-localisation function in Imaris is used to determine the fraction of liposomes internalised in microglia and astrocytes.

### Perls Prussian blue staining (Iron staining)

To assess for the presence of free iron in the brain which indicates RBCs haemolysis, Perls Prussian blue stain was performed. First, the slides were acclimatised to room temperature for 20 minutes and then hydrated in distilled water. The slides were then immersed for 20 minutes in a fresh mixture of equal parts of 20 % hydrochloric acid and potassium ferrocyanide. The slides were then washed in distilled water three times and then counterstained with nuclear fast red for 5 minutes and rinsed twice in distilled water. The slides were then dehydrated through 95 % alcohol once followed by 100 % alcohol two times over 3 minutes in each step. We cleared the slides in clear xylene twice (3 minutes each) and coverslip the slides using resinous mounting.

### MRI

Mice were imaged with MRI at 3 h, 24 h, 48 h, and 72 h following ICH. Not all mice received scans at each timepoint due to failed cannulation and spontaneous deaths - details of scanning time points for each mouse are given in supplementary materials. Before MRI, mice underwent tail vein cannulation under 4-5 % isoflurane in a mixture of 30 % oxygen and 70 % nitrous oxide for contrast agent administration. Scans were acquired on an Agilent 7T 16 cm bore magnet interfaced with a Bruker Avance III console running Paravision 6.0.1. During scanning, mice were anaesthetized using 2 % isoflurane in a mixture of 30 % oxygen and 70 % nitrous oxide. High-resolution axial T2-TurboRARE images were acquired for ventricle size assessment and definition of lesion ROIs. Acquisition parameters were: TR/TE = 3592/35 ms, NEX = 2, RARE factor = 8, echo spacing = 11.6 ms, matrix size = 256 x 256, 24 slices, slice thickness = 1 mm, field of view (FOV) = 16 x 16 mm. Axial 3D RF-spoiled gradient-echo images were acquired for *T*_1_ mapping (using the variable flip angle approach) and assessment of *T*_2_* signal changes. For *T*_1_ mapping the following acquisition parameters were used: TR/TE = 100 / 2.4 ms, flip angles = 5, 10, 40, 60, matrix size = 96 x 96 x 64, FOV = 16 x 16 x 24 mm. To quantify *T*_2_* signal changes, the spoiled gradient echo scan with a flip angle of 40° was repeated with a longer TE to increase the sensitivity of the scan to iron-based susceptibility gradients (TE = 4.95 ms). To quantify the leakage rate of contrast agent (*K*^trans^, [min-1]) across the blood-brain barrier, dynamic *T*_1_-weighted volumes were acquired before, during, and after intravenous injection of Gd-DOTA (0.5 M delivered through a tail vein cannula in a volume of 3.3 µL / g at a rate of 0.25 mL / min). The total duration of the dynamic scan was 40 minutes. Acquisition parameters were: TR/TE = 50/2.4 ms, flip angle = 60°, matrix size = 96 x 96 x 64, FOV = 16 x 16 x 24 mm. Details of MRI scan time points for each mouse are shown in Supplementary Table 1.

### MRI analysis

Ventricle and lesion regions of interest (ROIs) were manually delineated on the T2-Turbo RARE images using MRIcro (version 1.0.2). Ventricle volumes were calculated for each mouse by multiplying the number of voxels in each ROI by the voxel volume. *T*_1_ maps were generated by fitting the RF-spoiled gradient echo signal equation to measured voxelwise signal intensities as a function of flip angle. Voxelwise signal-time series from dynamic *T*_1_-weighted volumes were converted to contrast agent (CA) concentration time series, assuming a linear relationship between 1/*T*_1_ and CA concentration, and a spin-lattice relaxivity r_1_ = 3.5 (s mm)^-1^. Vascular input functions were measured by selecting a voxel in the superior sagittal sinus (SSS) as follows: the area under the concentration-time curve (AUC) for the first 5 post-contrast time points was calculated. The ROI was defined as the SSS voxel with a maximum AUC 79. *K*^trans^ maps were then generated by fitting the Patlak model to CA time series 80. All model fitting was done using 'lsqcurvefit' optimiser in Matlab (Mathworks, version 2017a).

To evaluate serial changes in *T*_1_, *T*_2_* signal, and *K*^trans^ in the same mouse, parameter maps for a particular mouse were co-registered to the map from the earliest imaging timepoint. This was done using in-house image registration software written in Matlab (Mathworks, release 2017a) based on maximising the product of the source and target local image gradients. The target image was defined as the first acquired SPGR volume (flip angle = 60°) and the source image as the matching SPGR volume from any subsequent timepoint. The estimated spatial transformations were then applied to the associated parameter maps. Visual checks of the aligned maps were completed to confirm successful registration. Once aligned, lesion ROIs defined on T_2_-RARE volumes were re-gridded to the matrix size of parameter maps. To quantify *T*_1_, *T*_2_* signal and *K*^trans^ changes, mean values in the lesion ROIs were calculated. To investigate areas of highest contrast agent leakage, the 95^th^ percentile of *K*^trans^ was also calculated.

### Statistical analyses

Statistical analysis of all data except MRI was performed using Graph Pad Prism 8 software. Two-tailed unpaired student t-test and one-way analysis of variance followed by the Tukey multiple comparison tests were used and p values < 0.05 were considered significant. MRI data statistical analysis was performed in R (version 4.0.2). Mixed-effects model analysis was performed instead of a one-way analysis of variance since not all mice were imaged at all timepoints. Mixed-effects model analysis was followed by Tukey multiple comparison tests and p values < 0.05 were considered significant. For all analyses, data are represented as mean ± standard error of the mean (SEM), unless otherwise indicated.

## Supplementary Material

Supplementary figures and table.
