# Supplementary material for: Selective brain entry of lipid nanoparticles in haemorrhagic stroke is linked to biphasic blood-brain barrier disruption
Source: Theranostics. 2022 May 26;12(10):4477–97. doi: 10.7150/thno.72167 (PMC9254235; doi:10.7150/thno.72167)
Supplement: Supplementary file 1 — Supplementary figures and table. [file thnov12p4477s1.pdf]

Selective brain entry of lipid nanoparticles in haemorrhagic stroke is linked to biphasic blood-brain barrier disruption

Authors: Zahraa S. Al-Ahmady[1,2#*], Ben R. Dickie[3,7#], Isabelle Aldred[2], Dhifaf A. Jasim[2], Jack Barrington[3], Michael Haley[3], Eloise Lemarchand[3], Graham Coutts[3], Satinderdeep Kaur[1], Jessica Bates[1], Sarah Curran[1], Ruth Goddard[1], Megan Walker[1], Adrian Parry-jones[4,5,6], Kostas Kostarelos[2*], Stuart M. Allan[3,6*]

Affiliations:

[1] Pharmacology Department, School of Science and Technology, Nottingham Trent University, Nottingham, NG11 8NS

[2] Division of Pharmacy and Optometry, Faculty of Biology, Medicine and Health, AV Hill Building, The University of Manchester, Manchester M13 9PT, United Kingdom.

[3]Division of Neuroscience and Experimental Psychology, School of Biological Sciences, Faculty of Biology, Medicine and Health, University of Manchester, Manchester, M13 9PT.

[4]Division of Cardiovascular Sciences, Lydia Becker Institute of Immunology and Inflammation, School of Medical Sciences, Faculty of Biology, Medicine and Health, Manchester Academic Health Science Centre, The University of Manchester, Manchester, UK

[5]Manchester Centre for Clinical Neurosciences, Salford Royal NHS Foundation Trust, Manchester Academic Health Science Centre, Salford, UK

[6]Geoffrey Jefferson Brain Research Centre, The Manchester Academic Health Science Centre, Northern Care Alliance NHS Group, University of Manchester

[#] Shared co-first authorship.

Supporting information

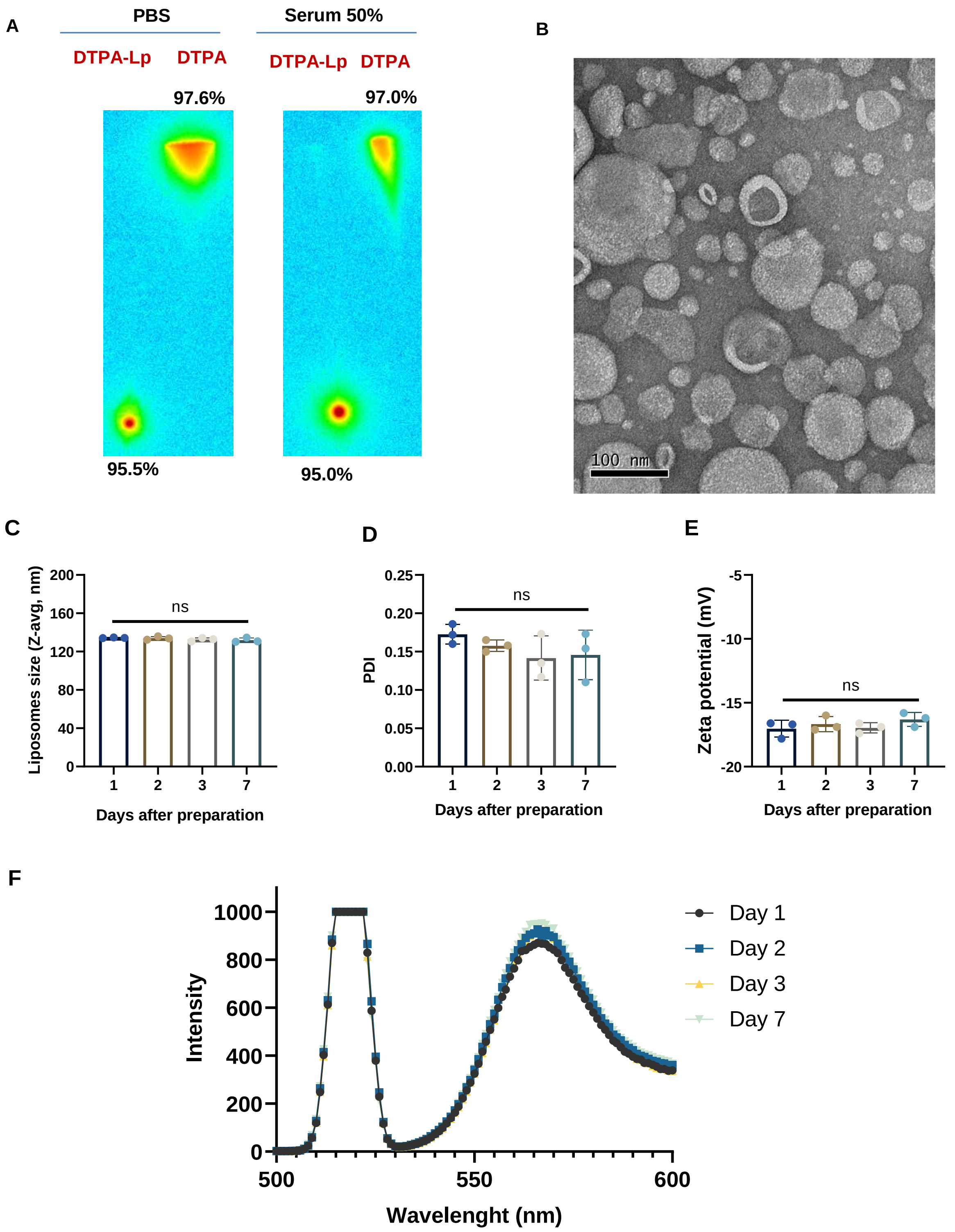

Figure S1: Characterisation data of liposomal formulations used in the study. A) Quantification of ^{111}In-Lp radiolabelling efficiency and stability in PBS and 50% serum by

TLC. The immobile spot on the TLC strips indicated the percentage of radiolabelled ^{111}In-Lp (~95%), while free ^{111}In was detected as the mobile spots near the solvent front (~97%). Very minimum free ^{111}In was detected in the ^{111}In-Lp samples in both PBS and 50% serum over 48 h period which confirmed the stability of radiolabelling. Radiolabelling stability studies were performed at 37°C to mimic physiological conditions. B) fluorescent intensity of DiI-Lp at the excitation wavelength of 518nm and emission wavelength of 565 nm. C) hydrodynamic diameter and D) zeta potential measurements of the liposomes indicating a size < 120 nm and slightly negative surface charge. E) TEM images of the liposomes confirmed the size range of the liposomes measured in DLS.

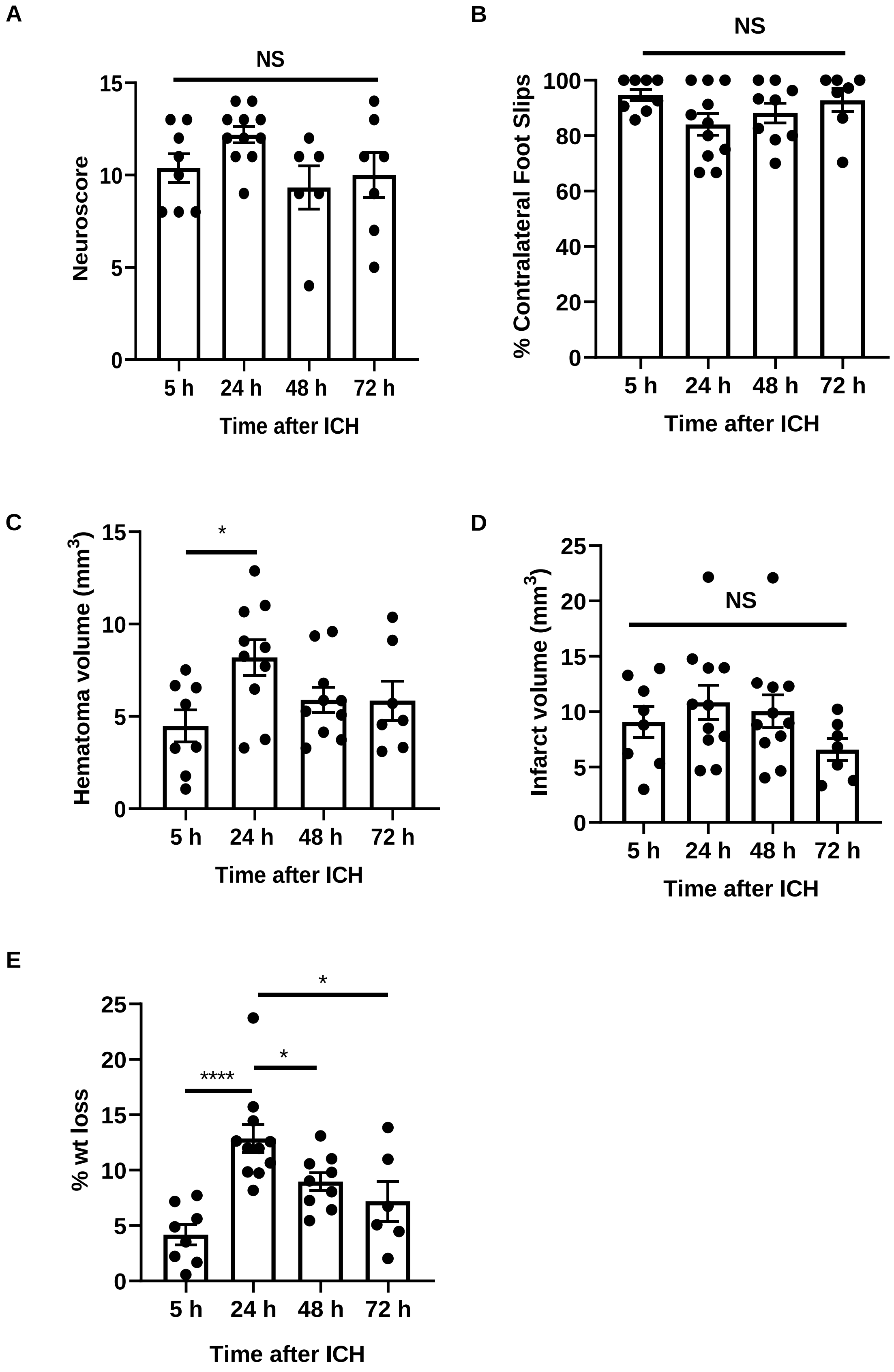

A
NS
Neuroscore
15
10
5
0
5 h
24 h
48 h
72 h
Time after ICH
B
NS
% Contralateral Foot Slips
100
80
60
40
20
0
5 h
24 h
48 h
72 h
Time after ICH
C
*
Hematoma volume (mm3)
15
10
5
0
5 h
24 h
48 h
72 h
Time after ICH
D
NS
Infarct volume (mm3)
25
20
15
10
5
0
5 h
24 h
48 h
72 h
Time after ICH
E
****
*
*
% wt loss
25
20
15
10
5
0
5 h
24 h
48 h
72 h
Time after ICH

Figure S2: Assessment of the behaviour function, histological brain changes and weight loss at different time points following induction of ICH model by collagenase injection. Assessment of focal brain damage induced by ICH was assessed by A) neuroscore system (0-28) and B) foot-fault test which confirmed the model and indicated no significant changes in these parameters over the 72 h tested. Histological analysis of the brain after ICH was performed by both C) H&E stain and D) Cresyl violet stain. which were used to quantify hematoma volume and infarct volume respectively on representative sections taken at eight defined coronal levels of brain tissues in each case. E) Mice general well-being was monitored by continuous measurements of body weight. Data indicated that the mice lost proximately 10% of the body weight in the 1st 24 h after ICH and recover quickly over that. Statistical analysis of C was performed using one-way ANOVA followed by the Tukey multiple comparison test and p values < 0.05 were considered significant.

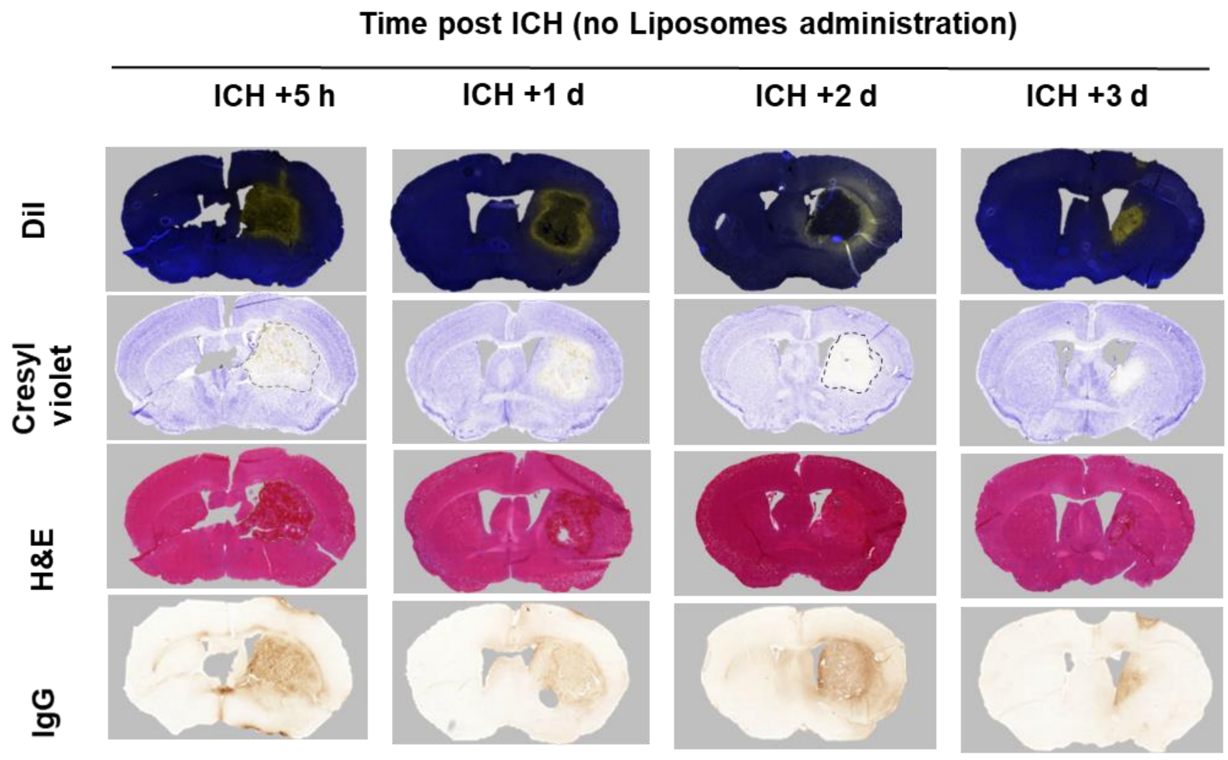

Figure S3: Histological analysis of brain tissues of ICH mice at different time points after ICH without injection of DiI-Lp confirmed that minimum autofluorescence signal (yellow signal) was detected in the injury site due to the presence of RBCs in the brain and tissue damage. This was found co-localised with the region of brain damage (represented by pale region with cresyl violet stain) and hematoma region as shown with H&E. IgG stain was performed to evaluate any endogenous IgG leakage into the brain that is used as an indication of BBB disruption. Statistical analysis was performed using ANOVA followed by the Tukey multiple comparison test and p values < 0.05 were considered significant.

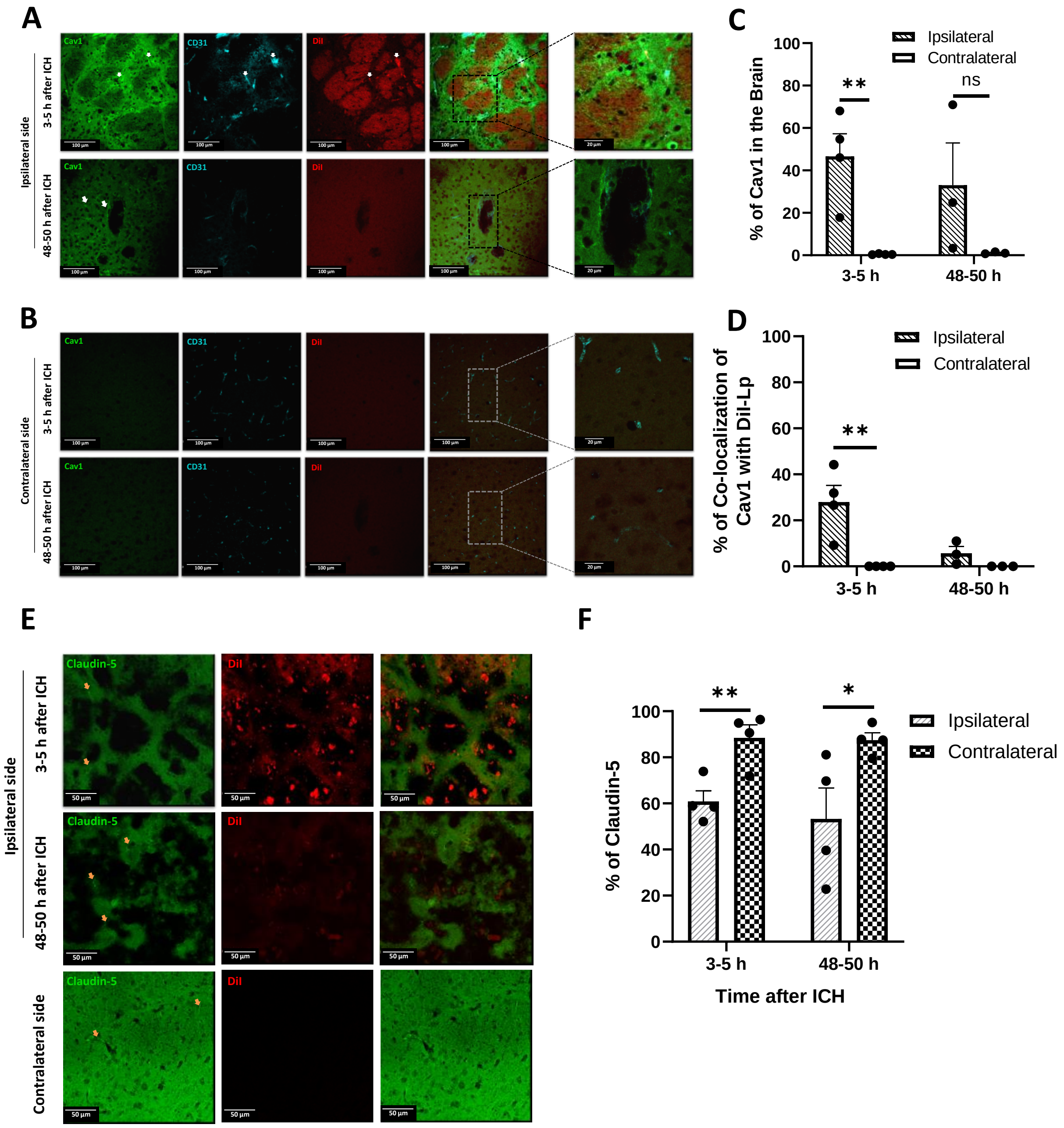

Figure S4: Representative confocal images of (A) ipsilateral and (B) contralateral side of brain tissues after ICH, suggested enhanced Cav1 positive area 3-5 h after ICH (indicated by white arrows) and showed a clear co-localisation with DiI-Lp positive areas. (C) quantification of Cav1 and (D) DiI-Lp positive areas showed a significant increase 3-5 h after ICH which suggest a role of transcytotic transport in the accumulation of liposomes into the lesion site after ICH. Cav1 expression 48-50 h after ICH was also higher than the contralateral side, however, this was not significant. (E) IHC staining for Claudin-5 to

evaluate changes to endothelial cells TJs showed a clear reduction in Claudin-5 positive regions in the ipsilateral side at both 3-5 h and 48-50 h after ICH compared to the contralateral side (orange arrows). (F) quantification of Claudin-5 positive signal confirmed a significant reduction in claudin-5 which suggest a role in TJ disassembly in the accumulation of liposomes at those time points.

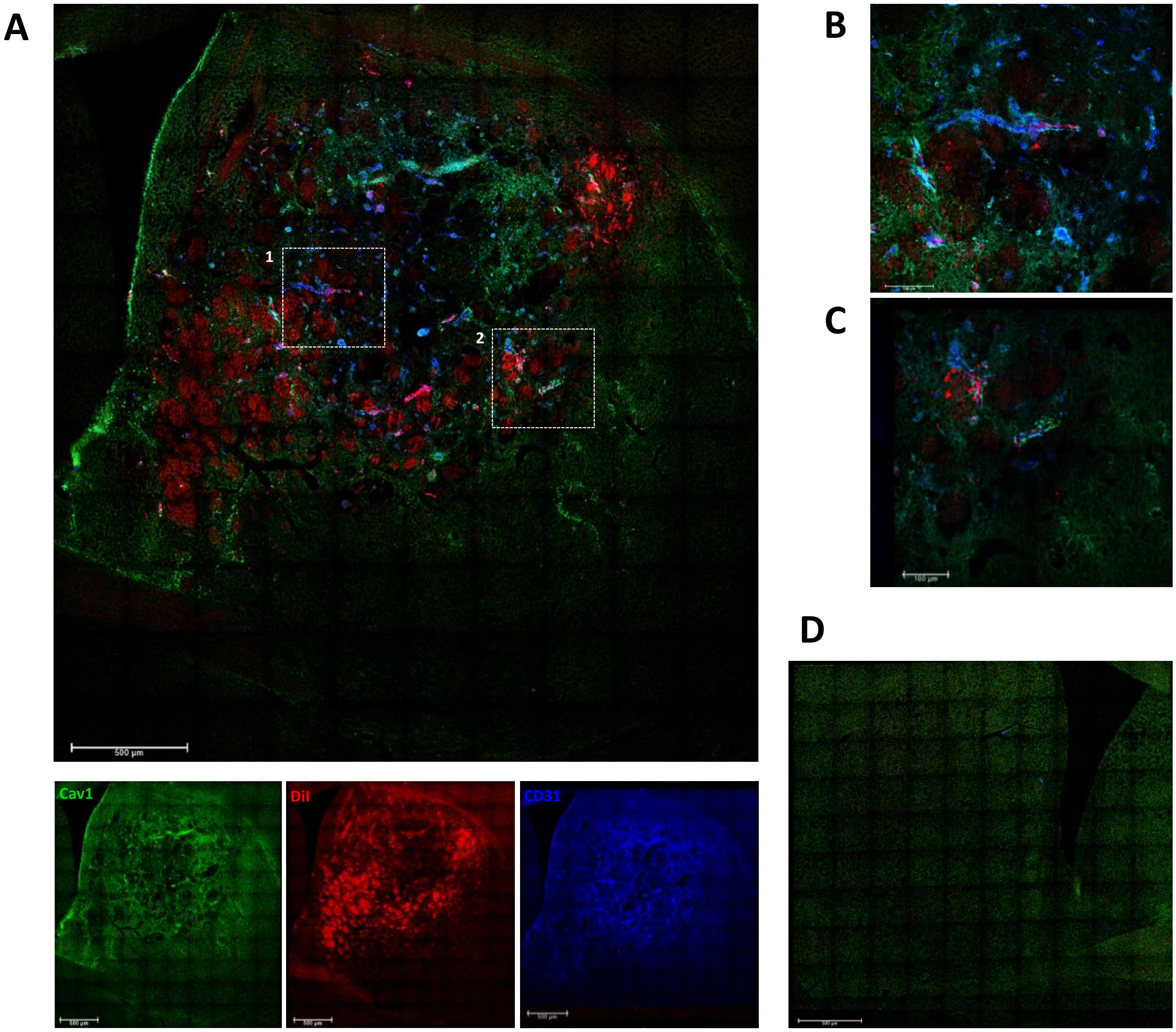

Figure S5: Representative widefield overview images were obtained at x40 magnification without oil utilising Leica Thunder imager microscope. All tissue areas were processed under identical conditions to demonstrate CAV1 (green), liposomes (red) and CD31 (blue) immunoreactivity. After initial imaging concluded, mosaic merge processing via Leica application suite X v3.7.4.23463 combined tiles into one image and large volume

computational clearing, such as lightning and thunder programme, reduced out of focus areas to produce high-quality images. Images represent (A) ipsilateral lesion, (B) ipsilateral core, (C) ipsilateral rim and (D) contralateral side of brain tissues taken 3-5 h after ICH and suggest enhanced Cav1 positive areas and show a clear co-localisation with DiI-Lp positive areas.

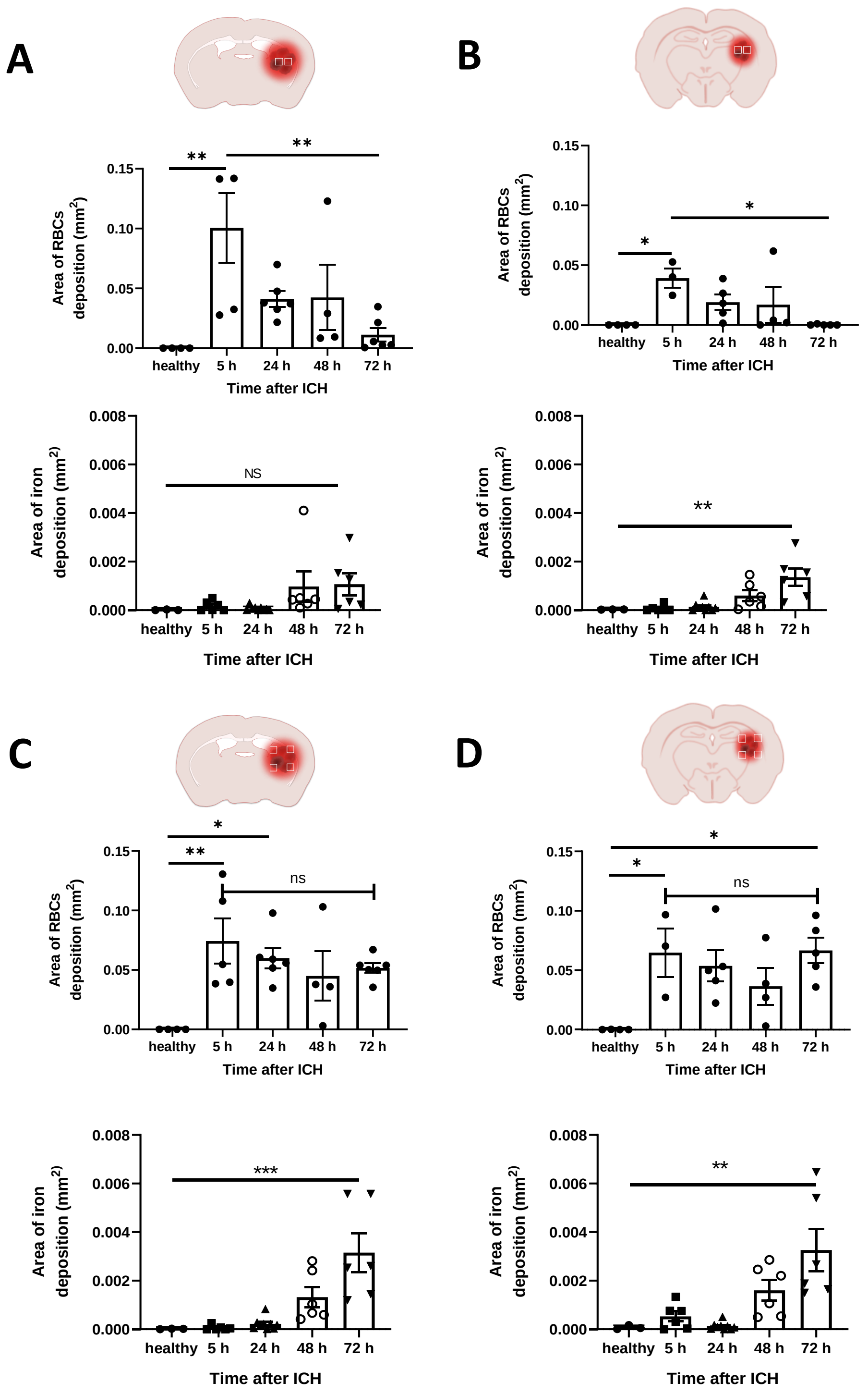

Figure S6 Histological analysis of iron deposition in the n after ICH using H&E and Prussian Blue stain on two different coordinates from the bregma; -0.58 (A&C) and -0.122 (B&D) both in the lesion core (A-B) and lesion rim (C-D). At each brain section measurements of positive RBC areas and positive free iron, stains were performed on

four rim regions and two core regions. H&E images indicated RBCs positive area decreased over time in the lesion core while the values are not significantly changed in the lesion rim. Prussian Blue stain on the other hand indicated a continuous rise in free iron as a result of RBCs haemolysis reaching significant levels around 72 h after ICH. Values are expressed as mean ± SEM. Statistical analysis was performed using one-way analysis of variance followed by the Tukey multiple comparison test and p values < 0.05 were considered significant.

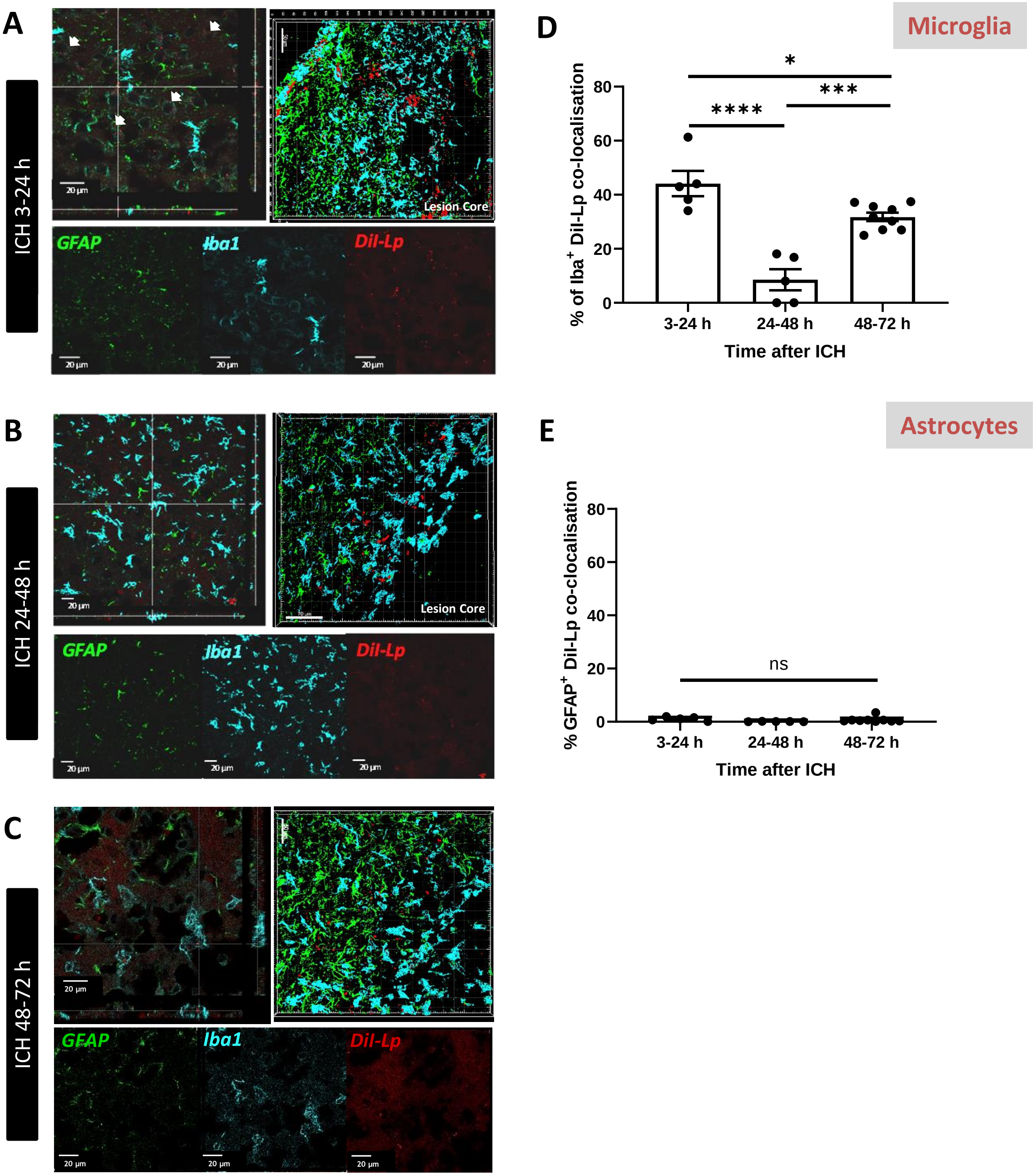

Figure S7: Evaluation of DiI-Lp co-localisation with microglia and astrocytes. Representative confocal images were taken 24 h after DiI-Lp injection into ICH at A) 3 h, B) 24 h and C) 48 h after inducing ICH with collagenase model. Images were analysed with IMARIS software to quantify the colocalization of liposomes with D) Iba1 (activated microglial marker) and E) GFAP (astrocytes marker). Quantification of DiI-Lp co-

localisations indicated significant uptake of DiI-Lp by microglia when injected 3 h and 48 h after ICH. This is also indicated by a white arrow on the confocal images. (G) No significant uptake of DiI-Lp was detected in astrocytes at all time points. Statistical analysis of the data in D&E was performed using one-way ANOVA followed by the Tukey multiple comparison tests, n=3-4 in each group. P values <0.05 were considered significant.

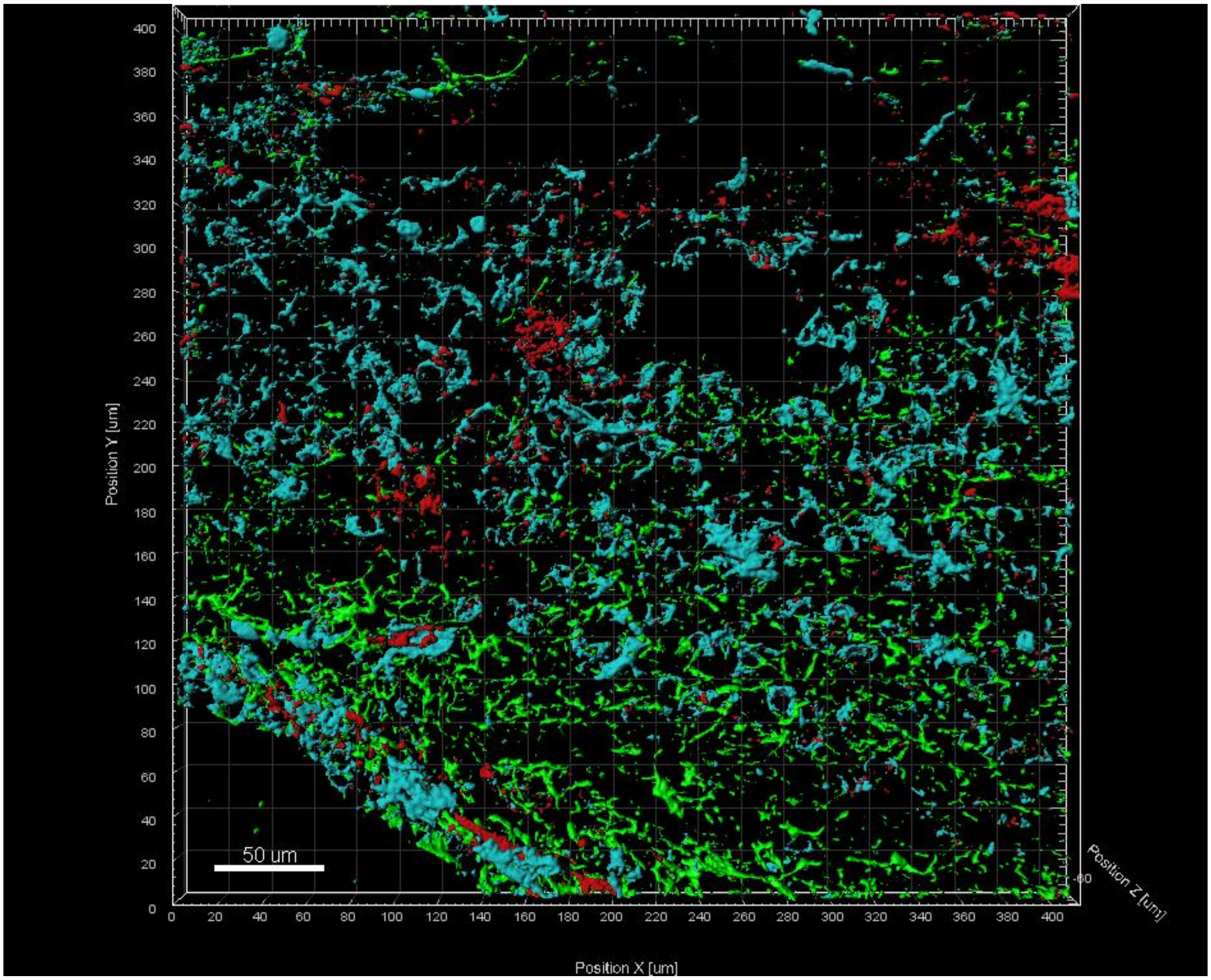

Figure S8: representative image analysis using Imaris software. Using Imaris software masks are created over each channel corresponding to liposomes, microglia and astrocytes. The image showing liposomes (red) colocalised mainly with microglia (cyan), while limited interaction was observed with astrocytes (green).

	3 h MRI (n = 7)	24 h MRI (n = 5)	48 h MRI (n = 5)	72 h MRI (n = 4)
**ICH mouse 1**	X			
**ICH mouse 2**	X	X	X	X
**ICH mouse 3**	X			

**ICH mouse 4**	X	X	X	X
**ICH mouse 5**	X	X	X	X
**ICH mouse 6**		X	X	X
**ICH mouse 7**	X			
**ICH mouse 8**	X	X	X	

	**3 h MRI (n = 2)**	**24 h MRI (n = 2)**
**Sham mouse 1**	X	X
**Sham mouse 2**	X	X

Supplementary Table 1. Details of MRI scan time points for each mouse. In instances where mice were not scanned at all timepoints, this was either because of failed cannulation (n = 1 – mouse 6, 3 h) or because the mouse died (mouse 1, 3, 7)**.** Additional DCE-MRI scans were performed in a 2nd cohort to confirm K^{trans} results and were performed only at 24 h and 72 h post-ICH (n = 7).
